# Supplementary figures and images for: A pilot phase Ib/II study of whole-lung low dose radiation therapy (LDRT) for the treatment of severe COVID-19 pneumonia: First experience from Africa
Source: PLoS One. 2022 Jul 1;17(7):e0270594. doi: 10.1371/journal.pone.0270594 (PMC9249221; doi:10.1371/journal.pone.0270594)

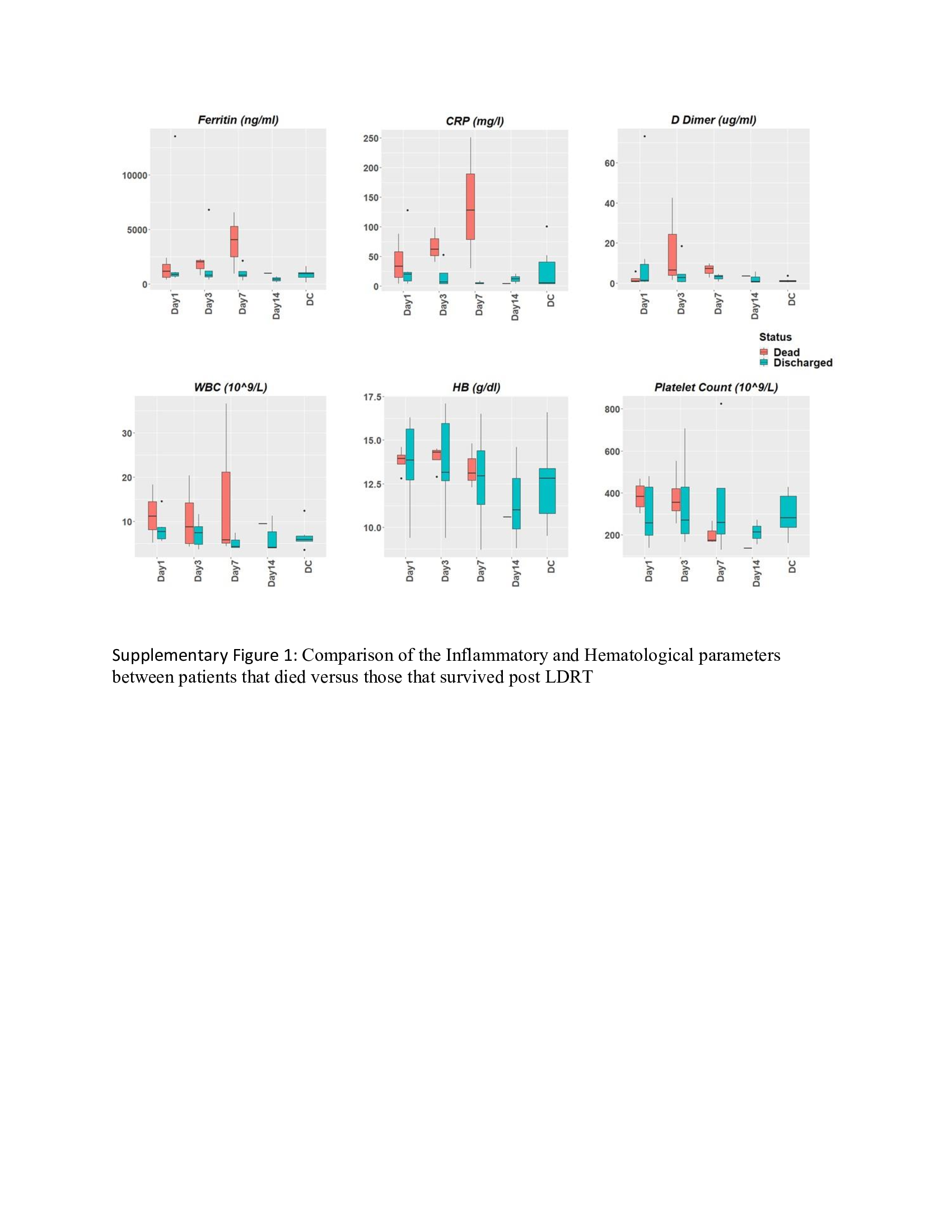

Supplement: S1 Fig — (TIF) [file pone.0270594.s002.tif]
