# Supplementary material for: A pilot phase Ib/II study of whole-lung low dose radiation therapy (LDRT) for the treatment of severe COVID-19 pneumonia: First experience from Africa
Source: PLoS One. 2022 Jul 1;17(7):e0270594. doi: 10.1371/journal.pone.0270594 (PMC9249221; doi:10.1371/journal.pone.0270594)
Supplement: S1 Protocol — (DOCX) [file pone.0270594.s003.docx]

**A pilot phase Ib/II study of whole-lung low dose radiation therapy (LDRT) in the treatment of severe Covid 19 pneumonia patients on (or requiring) mechanical ventilatory support.**

Principal Investigator Dr Mansoor Saleh, ^1^

Co-Principal Investigator Dr Farrok Karsan,^1^

Co-Investigator Dr Angela Waweru,^1^

Co-Investigator Dr Reena Shah,^2^

Co-Investigator Dr Martin Musumbi,^2^

1. Aga Khan University Hospital, Nairobi - Department of Haematology-Oncology
2. Aga Khan University Hospital, Nairobi - Department of Medicine.

**PROTOCOL NUMBER** Cov-LDRT

**SITE IDENTIFIER**

Aga Khan University Hospital Nairobi 3rd Parklands Avenue, off Limuru Road,

P 0. Box 30270, GPO 00100, Nairobi, Kenya

Telephone: +254 20 366 2107/2109; Fax: +254 20 374 4035

**STUDY IRB**

Aga Khan University, Nairobi Institutional Ethics Review Committee,

3rd Parklands Avenue, off Limuru Road,

Telephone +254 20 366 2148/+254 20 366 1136

**Protocol Version v4 dated 26Oct2020**

Contents

[**1.** **PROTOCOL SUMMARY** 6](#_Toc49200870)

[1.1. **SYNOPSIS** 6](#_Toc49200871)

[1.2. **Schema/Schedule of events** 8](#_Toc49200872)

[**2.** **INTRODUCTION** 10](#_Toc49200873)

[**3.** **BACKGROUND** 11](#_Toc49200874)

[3.1. Covid 19 Pneumonia: 11](#_Toc49200875)

[3.2. ARDS in Covid 19 pneumonia: 11](#_Toc49200876)

[3.3. Low dose radiation therapy (LDRT): 12](#_Toc49200877)

[**4.** **RISK/BENEFIT ASSESSMENT** 12](#_Toc49200878)

[4.1. **Exacerbation of existing covid-19 sequelae** 12](#_Toc49200879)

[4.1.1. Table 2: Total and fraction doses used in various forms of radiation therapy to the lung: 13](#_Toc49200880)

[4.1.2. Symptoms 13](#_Toc49200881)

[4.2. **Inherent side-effects of low-dose radiation therapy are low** 13](#_Toc49200882)

[4.2.1. Adverse Outcomes 13](#_Toc49200883)

[4.3. **Known potential benefits** 15](#_Toc49200884)

[4.4. **Risk mitigation strategies** 15](#_Toc49200885)

[4.4.1. Safe transfer of patients in compliance with hospital infection control guidelines 15](#_Toc49200886)

[4.4.2. Treatment logistics in compliance with hospital infection control guidelines 16](#_Toc49200887)

[4.4.3. Pre and post treatment observation 16](#_Toc49200888)

[**5.** **OBJECTIVES AND ENDPOINTS** 17](#_Toc49200889)

[**5.1.1.** **Primary Objective** 17](#_Toc49200890)

[**5.1.2.** **Secondary Objectives** 17](#_Toc49200891)

[**5.1.3.** **Primary Endpoint** 17](#_Toc49200892)

[**5.1.4.** **Secondary Endpoint** 17](#_Toc49200893)

[**5.1.5.** **Exploratory Endpoint** 17](#_Toc49200894)

[**6.** **STUDY DESIGN** 18](#_Toc49200895)

[**6.1.1.** **Overall design** 18](#_Toc49200896)

[**6.1.2.** **Data Safety Monitoring Committee (DSMC)** 19](#_Toc49200897)

[**6.1.3.** **Scientific rationale for study design** 19](#_Toc49200898)

[**6.1.3.1.** **Radiation Therapy for Benign Diseases (Including LCH and HLH) Relevance to Covid 19 Pneumonia and ARDS.** 19](#_Toc49200899)

[**6.1.4.** **Justification for dose** 20](#_Toc49200900)

[**6.1.5.** **Justification for the separate cohorts** 20](#_Toc49200901)

[**6.1.6.** **Safety Monitoring** 21](#_Toc49200902)

[**6.1.7.** **End of study definition** 21](#_Toc49200903)

[**7.** **ADVERSE EVENTS** 21](#_Toc49200904)

[**8.** **STUDY POPULATION** 22](#_Toc49200905)

[**8.1.1.** **Inclusion criteria** 22](#_Toc49200906)

[**8.1.2.** **Exclusion Criteria** 23](#_Toc49200907)

[9. **STUDY SITE** 23](#_Toc49200908)

[9.1. **Aga Khan University Hospital, Nairobi** 23](#_Toc49200909)

[9.2. **Geographical Location** 24](#_Toc49200910)

[9.3. **Language** 24](#_Toc49200911)

[9.4. **Radiotherapy (RT) facilities** 24](#_Toc49200912)

[9.5. **The Radiotherapy staff.** 25](#_Toc49200913)

[9.6. **Clinical Research Unit:** 25](#_Toc49200914)

[**10.** **BEST PRACTICE MANAGEMENT OF COVID 19 PNEUMONIA** 26](#_Toc49200915)

[10.1. **Management of COVID-19 at AKUH, N** 26](#_Toc49200916)

[**11.** **RECRUITMENT AND INFORMED CONSENT** 28](#_Toc49200917)

[**12.** **STUDY PROCEDURES** 29](#_Toc49200918)

[12.1. Screening 29](#_Toc49200919)

[12.2. Days 1, 3, 7, 14, and 28: 30](#_Toc49200920)

[12.3. Days 2, 4-6: 30](#_Toc49200921)

[12.4. Discharge 30](#_Toc49200922)

[**13.** **DATA MANAGEMENT** 32](#_Toc49200923)

[**14.** **CONFIDENTIALITY** 32](#_Toc49200924)

[**15.** **SAMPLE SIZE** 33](#_Toc49200925)

[**16.** **STATISTICAL ANALYSIS** 33](#_Toc49200926)

[**17.** **ETHICS** 33](#_Toc49200927)

[17.1. **Study Conduct** 33](#_Toc49200928)

[17.2. **Ethics Approval** 33](#_Toc49200929)

[17.3. **Protocol amendments** 34](#_Toc49200930)

[17.4. **Protocol deviations** 34](#_Toc49200931)

[17.5. **Serious adverse events reporting** 34](#_Toc49200932)

[17.6. **Unanticipated events** 35](#_Toc49200933)

[17.7. **Continuing Review Reports** 35](#_Toc49200934)

[17.8. **Inspections and Audits** 35](#_Toc49200935)

[17.9. **Data and Safety Monitoring Committee (DSMC)** 35](#_Toc49200936)

[**18.** **STUDY RELATED INJURY** 36](#_Toc49200937)

[**19.** **COMPENSATION** 36](#_Toc49200938)

[**20.** **DISSEMINATION OF RESULTS** 36](#_Toc49200939)

[**21.** **BUDGET** 36](#_Toc49200940)

[**22.** **REFERENCES** 37](#_Toc49200941)

[**23.** **APPENDICES** 40](#_Toc49200942)

[*23.1.* ***Appendix (i)*** *Informed Consent Form (English Version)* 41](#_Toc49200943)

[*23.2.* ***Appendix (ii)*** *Informed Consent form(Swahili)* 47](#_Toc49200944)

[*25.1.* ***Appendix (ii)*** *AKUH, N Inpatient COVID 19 management protocol* 53](#_Toc49200945)

[*25.2.* ***Appendix (iii)*** *Case Report Form* 54](#_Toc49200946)

# **LIST OF ABBREVIATIONS AND DEFINITIONS OF TERMS**

| AE | any untoward medical occurrence in a clinical investigation subject administered pharmaceutical product, regardless of causal attribution. |
| --- | --- |
| AKUHN | Aga Khan University Hospital , Nairobi |
| ARDS | Acute Respiratory Distress Syndrome |
| CCTV | Closed Circuit Television |
| cGY | a unit of absorbed radiation dose equal to one hundredth (10−2) of a gray |
| Cov | Coronavirus, Covid 19 |
| CRF | Case Report Form |
| CRP | C-Reactive Protein |
| CRU | Clinical Research Unit |
| CT Scan | computerized tomography (CT) scan |
| DSMC | Data Safety Management Committee |
| ECMO | Extracorporeal membrane oxygenation |
| FIO2 | fraction of inspired oxygen |
| GCP | Good Clinical Practice |
| HDU | High Dependency Unit |
| ICF | Informed Consent Form |
| ICH | International Council for Harmonisation |
| ICU | Intensive Care Unit |
| IERC | Institutional Ethics Review Committee |
| IP | Investigational Product |
| IRB | Institutional Review Board |
| LDRT | Low Dose Radiotherapy |
| LFT | Liver Function Test |
| MoH | Ministry of Health (Kenya) |
| PEEP | Positive end -expiratory pressure |
| PPE | Personal Protective Equipment |
| pre-vent | Pre- (Mechanical) Ventilation |
| RT | Radiotherapy |
| SARS | severe acute respiratory syndrome |
| SOC | Standard of Care |
| SUSAR | suspected unexpected serious adverse reactions |

# **PROTOCOL SUMMARY**

# **SYNOPSIS**

| Study description: | Hypothesis: Low-dose radiation therapy (LDRT) improves clinical, radiographic, and immune outcomes in hospitalized patients with covid-19 pneumonia and severe acute respiratory syndrome.  This study investigates LDRT as a selectively localized anti-inflammatory treatment to induce focal suppression of pulmonary immuno-toxicity within viral pneumonia infiltrates in hospitalized patients with COVID19.  Study: Administration of low dose whole lung radiotherapy (LDRT) for the treatment of severe COVID 19 pneumonia in patients on or requiring mechanical ventilation |
| --- | --- |
| Objectives: | To investigate safety and feasibility (primary endpoint) of low dose whole-lung radiation therapy (LDRT) in critically-ill patients with COVID-19 pneumonia, and to explore secondary efficacy endpoints: (1) clinical, (2) respiratory, and (3) outcome  **Primary Objective:**   1. To assess the feasibility and safety of administering LDRT to patients with severe Covid 19 pneumonia 2. To assess the toxicity of a single dose of LDRT administered to patients with severe Covid 19 pneumonia   **Secondary Objective:**   1. To assess improvement in oxygen requirement within 3 days following LDRT 2. To determine ability to come off mechanical (or avoid mechanical) ventilation within 7 days following LDRT 3. To determine time to discharge following LDRT 4. To determine time to death following LDRT |
| Endpoints | Primary   1. Safety/Feasibility – ability to complete LDRT without treatment related SAE 2. Toxicity – vital signs to assess clinical and respiratory status at 24h following LDRT   Secondary   1. Efficacy - ability to be weaned off pre-LDRT ventilatory or oxygen support at 3d post LDRT (% of patients) 2. Efficacy – ability to come off (or avoid) ventilatory support at 7d post LDRT (% of patients) 3. Efficacy - discharge or death by 14d / 28d post LDRT (% of patients) |
| SAMPLE SIZE | Up to 20 participants will be enrolled on this study.   - Cohort 1 (pre-Vent): Up to 10 participants - Cohort 2 (on-Vent): Up to 10 participants |
| Study population | 1. Patient with rtPCR and Xray/CT proven Covid 19 pneumonia who have not responded to conventional therapy and:   Cohort 1: would need mechanical ventilation if condition worsens (pre-Vent)  Cohort 2: has been on mechanical ventilation, but for not more than 5 days (on-Vent) |
| Phase: | Pilot Phase 1b/2 study |
| Description of sites/facilities enrolling patients: | 1. AKUHN – a collaborative effort of the Clinical Research Unit, Dept. of Haematology-Oncology (radiation therapy section), infectious diseases, critical care |
| Description of study intervention: | 1. Single dose of 100 cGy LDRT administered to both lungs within 48h of determining eligibility 2. Cohort 1 : 5 patients will be treated. If acute treatment related SAE is observed in ≤1 of 5 patients, additional 5 patients will be treated. 3. Cohort 2 : 5 patients will be treated. If acute treatment related SAE is observed in ≤1 of 5 patients, additional 5 patients will be treated.   If > 1 of the initial 5 patients in either cohort experiences acute treatment related SAE, enrollment to that cohort will be halted. |
| Study Duration: | 3 months |
| Participant duration: | 28d from enrollment |

# **Schema/Schedule of events**

**Table 1:**

| **Event** | | **Responsible Personnel** | **Screening**  **(complete within 7d prior to Treatment)** | **Day 1** | **Day 2** | **Day 3** | **Day 4** | **Day 5** | **Day 6** | **Day**  **7** | **Day 14/28** | **Discharge** |
| --- | --- | --- | --- | --- | --- | --- | --- | --- | --- | --- | --- | --- |
| Informed consent^1^ | | I | x |  |  |  |  |  |  |  |  |  |
| Medical History (SOC) | | I | x |  |  |  |  |  |  |  |  |  |
| Demographics (SOC) | | CRC | x |  |  |  |  |  |  |  |  |  |
| Screening/Registration | | I/CRC | x |  |  |  |  |  |  |  |  |  |
| Eligibility Confirmation | | I/CRC | x |  |  |  |  |  |  |  |  |  |
| Ordinal Score | | I/CRC | x | x | x | x |  |  |  | x | x | x |
| Vital Signs^2^ | | CRC | x | x | x | x | x | x | x | x | x | x |
| Routine labs (CBC+ diff, lytes, LFT, Ferritin, CRP, D-dimers) | | CRC | x | x |  | x |  |  |  | x | x | x |
| Special labs^3^ (stored) | | CRC | x | x |  | x |  |  |  | x | x | x |
| Oxygenation % and setting | | CRC | x | x | x | x | x | x | x | x | x | x |
| LDRT 100 cGy (single dose) | | I (RT) |  | x |  |  |  |  |  |  |  |  |
| Adverse Events^4^ | | I/CRC |  | x | x | x | x | x | x | x | x | x |
| Concomitant Medications^4^ | | CRC | x | x | x | x | x | x | x | x | x | x |
| Data entry | | CRC | x | x | x | x | x | x | x | x | x | x |
| PI Oversight | | I | x | x | x | x | x | x | x | x | x | x |
| Data Monitoring | | CRC | x | x | x | x | x | x | x | x | x | x |
|  | ^1^Informed consent to be provided by patient, or if unable to then to be provided by next of kin (closest first degree relative)  ^2^Vital signs (temperature, blood pressure, respiratory rate, pulse oximeter) if participant experiences an AE.  ^3^Blood sample for immune profile and cytokine measurements (stored) (IL-2, IL-7, granulocyte colony stimulating factor, interferon-γ inducible protein 10, monocyte chemoattractant protein 1, macrophage inflammatory protein 1-α, and tumor necrosis factor-α, ferritin, IL-6, IL-10, IFN- α, IFN- γ) at the following time points: Day 1, 3,7, 14, 28  ^4^Particpants discharged prior to Day 14 will continue to have safety monitoring (Adverse Events and Concomitant Medications) through Day 14. This may occur as a phone call. | | | | | | | | | | | |

*Fig 1*

# **INTRODUCTION**

Main Points

- Mortality from cascading inflammatory lung injury for intubated COVID-19 patients is 50-80%.
- Immuno-modulating LDRT eliminates cytokine storms and cured past pneumonias.
- There is precedent to use RT to treat a wide breadth of benign or inflammatory conditions.
- COVID-19 injurious mechanisms mimic histiocytosis, which has LDRT response rates of >90%.
- Radiation is routinely used in modern days to treat a histiocytosis disorder, especially the langerhan’s cell subtype. We suspect, it should also help with the HLH subtype that occurs in COVID-19 patients.

The novel coronavirus in 2019 (COVID-19) is associated with severe acute respiratory syndrome (SARS), intensive-care unit admission, and high mortality.^1,2^ There is little awareness among modern investigators that low doses of ionizing radiation therapy (LDRT) were successfully used to induced localized anti-inflammatory states as treatment of various types of infectious processes during the first half of the 20th century, including pneumonia, sinus infection, and skin infection.^3-5^ Anecdotally, RT reduced inflammation and reversed clinical decline at considerably lower doses than are currently used for oncologic purposes.^3^ Abruptly in the early 1940’s and for ensuing decades, the efficacy of RT in treating pneumonia and other infectious processes was eclipsed by the emergence of widely-successful anti-infectious drugs.^3^ In 2020, however, in an era of increasing antibiotic resistance,^6^ a seemingly-unstoppable viral killer has emerged. The SARS from coronavirus in 2019 (SARS-COVID-19) pandemic has shut down the global economy.^7,8^ COVID 19 is thought to induced a hyper-immune cytokine storm causing thoracic organ damage and unchecked edema through a pathway of accelerated immune activation. The overall mortality rate documented Covid 19 infection is 2-3%. On the other hand, 50-80% of severe cases requiring ventilation support lose their lives. Bilateral patchy ground glass opacities have been reported in essentially all such patients with 97% diagnostic sensitivity.^8-11^ In view of the alarming mortality seen with COVID-19, and the need to decrease intubation rates, our study proposes to re-evaluate LDRT as a localized anti-inflammatory treatment for COVID-19 pneumonia in critically-ill patients.

A number of therapeutic options have been shown to contribute to clinical improvement, including Remdesivir which inhibits viral shedding ^12^, Tocilizumab an monoclonal antibody directed against the IL-R receptor ^13.^ Both, as well as, dexamethasone^14^ may be beneficial at the onset of clinical worsening following O2 therapy^14^. Patients may also be eligible for convalescent plasma ^15^. Approx. 5-10 % of patient develop worsening symptoms and lack of adequate oxygenation due to the rapid development of a brisk immunologic response and the release of cytokines (cytokine storm)^16^. Such patients develop high oxygen requirement including need for high flow oxygen (which is generally not available at most LMIC institutions). Such patients have been shown to benefit from the introduction of steroids ^14^. Patients who do not respond to high flow oxygen in combination with the various treatments, often need to undergo intubation and mechanical ventilation as the last resort to protect adequate oxygenation and avoiding circulatory collapse. No specific treatment has been found to be beneficial in such patients and mortality can be as high as 80%.

Our study is based on the efficacy of LDRT over the past half a century in the treatment of inflammatory processes involving the lung, where low doses of RT have positively impacted outcome. With the understanding of the pathobiology of ARDS associated with Covid 19 cytokine storm, single LDRT has recently be successfully tried in patients with severe Covid 19 pneumonia ^17,^  ^18.^ and five clinical trials are currently active in N. America and Europe. No such studies are underway in Africa.

# **BACKGROUND**

# Covid 19 Pneumonia:

Pneumonia caused by a viral etiology provokes an inflammatory immune response causing the alveoli to become inflamed, compromising gaseous exchange. The immune cells triggered by the virus release pro inflammatory cytokines and chemokine such as IL-1 and IL-6. ^19^ Low dose radiation therapy has been used to treat pneumonia patients in the past, and multiple publications in the early nineteenth century covering over 700 pneumonia patients showed measurable improved clinical improvements within hours and days of administration ^20.^

Deaths caused by COVID-19 are characterized by acute respiratory distress syndrome (ARDS), pneumonia, sepsis and respiratory failure. Combined with the high transmission rate of the virus, the health care system has been overly burdened worldwide^9^. And while nearly a century later, the standard of care of pneumonia has changed, discovery of the mechanisms and pathways of LDRT’s immune-modulating effects can be applicable in the search for new strategies to reduce the burden of Covid-19.

# ARDS in Covid 19 pneumonia:

Acute Respiratory Distress Syndrome (ARDS) in COVID-19 patients appears to be induced by a hyper inflammatory syndrome similar to Secondary Hemophagocytic Lymphohistiocystosis (sHLH) or Macrophage Activation Syndrome (MAS).^21^ sHLH and MAS are cytokine storm-like syndromes most commonly associated with rheumatologic disorders,^22,23^ however, viral disease in both preclinical models and in the clinical setting have been shown to induce a similar phenomenon.^24,25^ Pre-clinical data has shown CD8+ T-cell dysfunction is critical for sHLH development following a viral infection with CD8+ T-cell secreted IFN-gamma being an important mediator.43 Mehta et al. noted that severe COVID-19 patients have a cytokine profile similar to sHLH characterized by increased IL-2, IL-6, and INF-gamma.40

# Low dose radiation therapy (LDRT):

Low dose RT has long been known to be efficacious in the treatment of several benign conditions including Graves orbitopathy, Morbus Dupuytren, and Peyronoies Disease. The use of radiation allows symptomatic relief with minimal toxicities in this cohort of patients. ^26,27-29, 30,31^ This favorable risk/benefit ratio in these low-acuity settings justifies the consideration of RT in patients suffering from higher-acuity disease processes, for whom the potential benefits are considerably higher, and the risks are lower. Multiple mechanisms of action have been proposed to explain the observed anti-inflammatory and anti-fibrotic effects of LDRT, including but not limited to lymphocyte depletion through ionizing damage, exudative reduction, and wavelength vibrations causing immune and pathogenic cell disruptions.^32,33^

Therefore, it is likely that radiotherapy may play a role in mitigating COVID-19’s HLH-mediated inflammatory storm and needs to be assessed in this pilot phase prospective trial. A recent study by colleagues at Emory University in Atlanta, GA have demonstrated the benefit and lack of toxicity of this modality in a group of severely ill Covid 19 patients ^17^ . A recently NCI virtual symposium underscored the potential benefit of this strategy and currently there are number efforts to study the basis for these favorable results and 5 studies have been registered with the NCI.GOV exploring various LDRT strategies in Covid 19 patients.

Unlike most studies, ours will be limited to those patients who desperately are in need for a therapeutic option to overcome the cytokine storm associated ARDS, having failed all available convention modalities and dependent of mechanical ventilation for life support.

# **RISK/BENEFIT ASSESSMENT**

# **Exacerbation of existing covid-19 sequelae**

Investigation into the safety of whole-lung low-dose radiation in critically-ill patients with COVID-19 requires prediction of potential interactions between low-dose ionization events and all previously-reported symptoms and adverse outcomes associated with COVID-19. Low-dose radiation therapy is a well-tolerated and well-studied treatment. 100cGy is unlikely to cause acute toxicity other than fatigue and transient cytopenia, and is much lower than what is currently being used for the treatment of malignancies the involved the lung (Table 2):

# Table 2: Total and fraction doses used in various forms of radiation therapy to the lung:

| **Disorder** | **Total dose** | **Fractions** |
| --- | --- | --- |
| Pediatric Wilms tumor – whole lung | 1500 - 1800 cGy | 150 cGy x 10-13 |
| TBI (BM transplant) – whole lung dose | 800 cGy | 200 cGy x 4 |
| Lung cancer- partial lung | 5000 – 6000 cGy | 200 cGy x 25 - 30 |
| Breast cancer – partial lung | 6000 cGy | 200 cGy x 30 |

# Symptoms

Fever, cough, myalgia, fatigue, sputum production, headache, hemoptysis, diarrhea, dyspnea have all been reported with COVID-19.^1^ Radiation therapy to the whole lung at a dose of 100cGy is not associated with acute toxicity, with the possible exception of fatigue. Radiation may exacerbate the pre-existing fatigue of COVID-19 patients. Otherwise, few acute toxicities are anticipated with low dose lung radiotherapy.

# **Inherent side-effects of low-dose radiation therapy are low**

# Adverse Outcomes

To date, the virus has been associated with high mortality, as well as adverse sequelae including but not limited to lymphopenia, leukocytosis, leukopenia, hypoalbuminemia, pneumonia, abnormal chest CT, acute cardiac injury, hypercoagulable state, acute respiratory distress syndrome, secondary infection, lung parenchyma destruction, diffuse alveolar damage, cardiomyopathy, brain injury, pleurisy, pericarditis, shock, and cytopenia.^1,34,35^

Lung irradiation is a well-studied treatment for both childhood and adult malignancies. Lung RT has well-established dosimetric threshold limits for the avoidance of toxicities such as radiation pneumonitis.^36,37^ Children with metastatic cancer such as Wilms tumor or Ewing sarcoma have been receiving whole-lung irradiation to doses of 1500-1800 cGy for decades, delivered in 150 cGy daily fractions over 10-13 treatments.^38^ The proposed single dose of 100 cGy proposed for our study is not expected to cause any acute or long term toxicity.

**The following are the potential organ systems affected by Covid 19, which may need special attention in the context of LDRT:**

1. *ARDS/Lung Parenchyma Destruction/Pulmonary Edema/ Pleurisy/Pneumothorax)*

Total-body irradiation (TBI) for marrow ablation is common in the setting of stem cell transplant, utilizing doses around 1200-1400 cGy, but again typically delivered in smaller fractions of 150-250 cGy per day with lung blocks to reduce pulmonary exposure.^39^ Safety parameters for single-fraction radiation therapy has, more recently, been established for palliation of lung metastases at dose ranges of around 800 cGy per single fraction for peripheral non-small lung cancers.^40,41^ Pneumonitis constraints for lung radiation treatments limit the total lung volume receiving 500 cGy to be 60% or less. The proposed single-fraction dose in this study of 100 cGy is considerably lower than any of these published lung toxicity thresholds.

1. *Cardiomyopathy/Myositis/Heart Failure/Pericarditis*

Patient with COVID-19 can develop cardiac injury diagnosed through elevated troponin levels.^1^ Evidence of this injury has been strongly correlated with mortality in initial studies of early disease cohorts from Wuhan, China.61 Mortality rates for those with underlying cardiovascular disease and elevated troponin levels is reported by numerous authors at 70%.^42,43^

Radiation therapy can induce late cardiac toxicity when given at doses of 4000cGy as administered to patient with breast cancer with the heart being adjacent. This is unlikely to occur at the doses suggested in the present study.

1. *Liver + Biliary Dysfunction*

Radiation-induced liver injury (RILD) is a rare late radiation toxicity typically avoided by liver constraints that preserve portions of the liver dose to 3000 cGy.^44^ Our proposed dose of 100 cGy may overlap with the upper portion or perhaps all of the liver, but our dose is 10-20 times lower than the published tolerance and thus unlikely to contribute significantly to COVID-related liver injury.

1. *Hypovolemic Shock//Cardiogenic Shock/Septic Shock*

There is increasing data that COVID-19 infection can lead to shock through a variety of mechanisms including cardiac injury, superinfection, and superimposed hypovolemia.66 Radiation therapy has little impact on the volume status or fluid balance and is unlikely to exacerbate this COVID sequelae.

1. *Cytopenia/Lymphocytopenia/Splenic atrophy^45^*

Radiation to the whole lungs at doses of 1800 cGy has the potential to exacerbate cytopenia, since thoracic radiation alone is known to induce lymphocytopenia and could worsen this outcome in COVID-19 patients.^46^ Decreased numbers of lymphocyte, cell degeneration and necrosis have been observed in the autopsy reports of spleens following lethal COVID-19 infection. The propensity for low-dose radiation therapy to focally deplete the immune repertoire is the mechanism objective of this protocol. Radiation to the lung has the capability of depleting lymphocyte stores in the adjacent spleen. This is not expected following a single dose of 100cGy to the lung.

# **Known potential benefits**

Historical precedence in regards to use of LDRT has been encouraging and has been the basis for the first trial of LDRT in patients with Covid 19 pneumonia ^17^ This has recently led to 5 additional trials studying various modification of LDRT in this population as well as an NIH Virtual Symposium on this important subject. Based upon one completed study, there has been no acute toxicity and remarkable outcome in some of the severely ill patients (personal communications, ^17^).

Our proposal to use this novel modality in severely ill patients who have limited or no conventional options provides a risk benefit ratio in favor of patient safety and benefit with minimal toxicity. Our study will provide data to demonstrate the ability of LMIC institution to provide this potentially lifesaving strategy in the setting of a surge in Kenya and lack of adequate ICU beds and ventilators.

# **Risk mitigation strategies**

At AKUHN we have had experience treating both Covid 19 patients (with cancer) for palliative RT while maintaining strict compliance with hospital infection control measures, as well as treating cancer patients who have been on ventilators. As such the experience and expertise to conduct this study is available at the AKUHN radiation therapy unit.

Delivery of external radiotherapy to COVID-19 positive patients is thus within the standard operating procedures of RT unit at AKUHN. However, there is risk of COVID-19 transmission to healthcare workers and contemporaneous COVID-19 negative individuals needs to be mitigated in the same way as is currently being done by other diagnostic departments e.g. radiology where Covid 19 patients need to be catered for. Strict adherence to the most up to date intuitional guidelines for distancing, personal protective equipment, and disinfection is critical and will be employed. To this end, the investigator team has been in consultation with hospital infection control to ensure compliance measures are in place.

# Safe transfer of patients in compliance with hospital infection control guidelines

1. PPE as per hospital policy for all staff involved in delivering protocol treatments
2. Transfer by stretcher
3. Patient cover will follow hospital infection control guidelines to limit aerosol transmission from the patient being transported with accompanying portable ventilator or oxygen delivery apparatus
4. Use of a single dedicated elevator to bring patient/s to the basement RT suite.
5. Elevator and stretcher will be sanitized following transportation in compliance with hospital infection control guidelines

# Treatment logistics in compliance with hospital infection control guidelines

1. COVID patients for trial to be treated after normal working hours
2. One Linac room will be dedicated for the treatment of all protocol patients (same on each treatment day)
3. Pre and post use cleaning and decontamination of RT suit as per hospital infection control protocol
4. Use of UVC light in the RT room after completion of treatments to further reduce risk of infection
5. All personnel and staff involved in the treatment/transportation/transfer of the patient will be in full PPE and will donn and doff per hospital infection control guidelines. All accessories and other mobile items to be removed from the Linac room
6. All surfaces to be covered by disposable plastic sheets – Linac couch, head of Linac, hand pendant, accessories used
7. Items for re-sterilization and re-use to be put in specially marked bag and left inside the Linac room
8. The Linac room is equipped with negative pressure for purposes of general radiotherapy with a 100% fresh air exchange.

# Pre and post treatment observation

1. Vital signs including SpO2 will be measured prior to start of LDRT and at the end of LDRT, prior to transport back to the unit and upon arrival at the unit.
2. Vital signs and clinical observation will be done hourly for the first 4h following treatment and then as per best practice clinical guidelines.
3. It would be desirable not to introduce new therapeutic intervention for 24 hours after radiotherapy unless medically necessary. However, clinicians taking care of the patients will be at absolute liberty to continue best practice management of all patients, whether on study or not.

# **OBJECTIVES AND ENDPOINTS**

# **Primary Objective**

1. To assess the feasibility and safety of administered LDRT to patients with severe Covid 19 pneumonia
2. To assess the toxicity of a single dose of LDRT administered to patients with severe Covid 19 pneumonia

# **Secondary Objectives**

1. To determine improvement in Oxygen requirement within 3 days following LDRT
2. To determine ability to come off mechanical ventilation within 7 days following LDRT
3. To determine time to discharge following LDRT
4. To determine time to death by following LDRT

# **Primary Endpoint**

1. Safety/Feasibility – % patients able to complete LDRT without treatment related SAE at 24h following LDRT
2. Toxicity – % patients without worsening of vital signs to assess clinical and respiratory status at 24h following LDRT (CTCAE acute toxicity criteria)

# **Secondary Endpoint**

1. Efficacy - % patient able to be weaned off pre-LDRT ventilatory or oxygen support at 3d post LDRT
2. Efficacy - % patients able to come off (or avoid) mechanical ventilation within 7 days following LDRT
3. Efficacy - % patients discharged or expired at 14d/28d post LDR

# **Exploratory Endpoint**

1. Immune response and cytokine profile will be studied retrospectively as part of “special send out tests/labs”

# **STUDY DESIGN**

# **Overall design**

This is a pilot Phase 1b/2 study to determine safety and feasibility of administering LDRT to the whole lung in a Covid 19 patient population who have progressed following administration of conventional therapy and are on ventilatory support or who are decompensating despite conventional therapy and are at risk of requiring ventilatory support to sustain life.

The rational basis is the effort to influence the pulmonary immune repertoire that is responsible for the cytokine storm responsible for the ARDS that leads to the need for mechanical ventilatory support in such patients. Previous experience at Emory University has been very favourable ^17^ Of the 10 patients (median age 78) who were requiring supplemental oxygen whose health was visibly deteriorating, received a single dose of 150 cGy. Patients were in and out of the RT unit within 10-15 minutes. Within 24h, 9/10 patients showed rapid improvement and were discharged within 12 days. No acute toxicity was observed (personal communication ahead of publication). In the meantime, there are 5 active trials studying the ability of various strategies of LDRT in Covid 19 patient population.

Our study determines to assess the safety and feasibility of administering LDRT in the LMIC setting in patients who may have different comorbidities than seen in the West, where extraordinarily expensive therapeutic agents may not be available, and where ventilators are in extremely short supply.

A single dose of LDRT is seen as a relatively inexpensive treatment in patients at the extreme end of the clinical spectrum of Covid 19 pneumonia (on ventilators or destined to need ventilator support).

Eligible patients will receive a single dose of LDRT of 100cGy which has been found to be safe and efficient is comparable settings. Patients will be followed for toxicity for the duration of their hospital stay with specific study related endpoints being determined at 24h, 3d, 7d, 14d, and 28d.

This will be a single arm, single dose pilot study involving 2 separate cohorts (Cohort 1: pre-Vent / Cohort 2: on-Vent). 5 patients will be initially enrolled on each cohort. If ≤ 1 patients experience an acute LDRT associated SAE, an additional 5 patients for a total of 10 will be enrolled.

Special labs will be collected from participants for future testing as biological correlates of response of progression.

The RT section at AKUHN has experience treating Covid 19 patients in full compliance with current infection control guidelines. In addition, we also have experience treating ventilated patients under urgent/emergent clinical setting and would thus be able to treat the patient population described in this protocol.

# **Data Safety Monitoring Committee (DSMC)**

A Data Safety Monitoring Committee (DSMC) composed of 3 individuals (clinician, clinical investigator, statistician) not involved in the study will be constituted that will review the outcome of each of the first 5 patients in each cohort at 24h following treatment. If >1 of the initial 5 patients experience an SAE felt to be related to LDRT by the investigator and/or DSMC, the study will be halted. If ≤ 1 patients experience a related SAE, a total of 10 patients in each cohort will be enrolled. Any SAE observed in any of the additional patients will be reported to the DSMC for review and discussion.

# **Scientific rationale for study design**

# **Radiation Therapy for Benign Diseases (Including LCH and HLH) Relevance to Covid 19 Pneumonia and ARDS.**

Low dose RT has long been known to be efficacious in the treatment of several benign conditions including Graves orbitopathy, Morbus Dupuytren, and Peyronoies Disease. The use of radiation allows symptomatic relief with minimal toxicities in this cohort of patients. ^47-49,28-33^

This favorable risk/benefit ratio in these low-acuity settings justifies the consideration of RT in patients suffering from higher-acuity disease processes, for whom the potential benefits are considerably higher, and the risks are certainly no greater.

Multiple mechanisms of action have been proposed to explain the observed anti-inflammatory and anti-fibrotic effects of LDRT, including but not limited to lymphocyte depletion through ionizing damage, exudative reduction, and wavelength vibrations causing immune and pathogenic cell disruptions.^3^

Acute Respiratory Distress Syndrome (ARDS) in COVID-19 patients appears to be induced by a hyper inflammatory syndrome similar to Secondary Hemophagocytic Lymphohistiocystosis (sHLH) or Macrophage Activation Syndrome (MAS).^21^ sHLH and MAS are cytokine storm-like syndromes most commonly associated with rheumatologic disorders,^22,23^ however, viral disease in both preclinical models and in the clinical setting have been shown to induce a similar phenomenon.^24,25^ Pre-clinical data has shown CD8+ T-cell dysfunction is critical for sHLH development following a viral infection with CD8+ T-cell secreted IFN-gamma being an important mediator.^24^ Mehta et al. noted that severe COVID-19 patients have a cytokine profile similar to sHLH characterized by increased IL-2, IL-6, and INF-gamma.^21^

Lymphocytes, a main mediator of sHLH, are extremely sensitive to ironizing radiation with a D90 (dose required to reduce the lymphocyte population to 90% of initial values) of 50cGy.^50^ Fractionated radiotherapy for intracranial malignancies has been shown to induce lymphopenia.^51^ Interestingly, the data evaluating pulmonary irradiation and inflammatory infiltrates is extremely dependent on the dose and timing. Paun et al. showed increased lymphocytic lung tissue infiltration at day 1 and 7 following 1800cGy to the whole thorax.^52^ On the other hand, Zheng et al. showed that 250 cGy TBI induces a delayed pulmonary inflammatory reconstitution with a CD8+ T-cell minima at 3 days post irradiation.^53^ Importantly Zheng et al. did not show a rebound increase in lung T-cell infiltrates following RT, suggesting that a low radiation dose may be effective at transiently lympho-depleting the lungs without inducing a pro-inflammatory rebound.

The probability that lymphoid-depleting LDRT will cause clinically significant reduction in the HLH-mediated cytokine storm in COVID-19 patients is most well-demonstrated in the published outcomes of patients irradiated for other histiocytic disorders. Among these, clinical experience with LDRT for the treatment of Langerhans Cell Histiocytosis (LCH) is most robust. Researchers report exceptional response rates of over 90% of patients with LCH. Complete responses range from 76% to 93% and the majority of the rest experienced at least a partial response following LDRT in doses typically ranging from 6 to 20 Gy.^54,55^ Non-LCH are comparatively rare, among which HLH is the next most common disease and is clinically characterized by hyper-activation of ordinary macrophages and lymphocytes leading to the systemic hyper-inflammatory syndrome as discussed before.^56^We speculate that if radiotherapy is this effective in treating LCH, it might also help reduce HLH in COVID-19 patients.

# **Justification for dose**

As discussed in the introduction, single doses of 50 – 200 cGy have been used over the past decades for the treatment of inflammatory immune conditions. This is compared to nearly higher doses being used for multiple days in the treatment of cancers or histiocytic disorders. Currently doses of 70 – 150 cGy are being studied across N. America and Europe. We have selected a dose of 100 cGy for our proposed study.

# **Justification for the separate cohorts**

It is unclear as to the optimal time to intervene with LDRT in the setting of cytokine storm and ARDS. Patients having been on ventilatory for a prolonged time period may have irreversible pulmonary damage and thus not respond to the anti-inflammatory effects of a single dose of LDRT and potential suffer toxicity. We thus plan to enrol 2 distinct cohorts of patients on this study so as to separate patients who may have irreversible lung damage (prolonged ventilation) from those who not yet been placed on a ventilator. Cohort 1 will be patients who have thus far been on various non invasive O2 supplementation (e.g. nasal canula, venti-marks, rebreather mask, non-rebreather mask), and their condition does not appear to be improving or may be worsening and are at risk of requiring intubation (pre-Vent). Cohort 2 will be those patients who are already on ventilatory support for a maximum of 5 days. Each cohort will enrol independently and assessed independently.

# **Safety Monitoring**

Low dose Radiation will be administered in in accordance according to the AKUHN SOPs on administration of Radiation. The pre, during and post dose monitoring details will be captured and documented. These include:

1. Vital signs including SpO2 will be measured prior to start of LDRT and at the end of LDRT, prior to transport back to the unit and upon arrival at the unit.
2. Vital signs and clinical observation will be done hourly for the first 4h following treatment and then as per best practice clinical guidelines.
3. Safety monitoring will be done daily for the fourteen (14) day observation period.

# **End of study definition**

All eligible patients following consent will receive a single dose of LDRT within 48h of meeting eligibility and being enrolled on the study. Patients will undergo routine physical examination, vital signs and oxygenation parameters on a daily basis as part of standard of care. Observation period for the study will end on day 28 or at the time of discharge (or death), whichever comes first.

# **ADVERSE EVENTS**

According to ICH Guideline for Good Clinical Practice, and for the purpose of this protocol, an adverse event is defined as any untoward medical occurrence in a clinical investigation subject administered pharmaceutical product, regardless of causal attribution. An adverse event can therefore be any of the following:

- Any unfavourable and unintended sign, symptom, or disease temporarily associated with these of a medicinal product whether or not considered related to the medicinal product.
- Any new disease or exacerbation of an existing disease (worsening in the character, frequency, or severity of a known condition)
- Recurrence of an intermittent medical condition not present at baseline.
- Any deterioration in a laboratory value or other clinical test that is associated with symptoms or leads to a change in the study treatment or concomitant treatment or discontinuation from Study drug.
- Adverse events that are related to protocol-mandated intervention, including those that occur prior to assignment of study treatment. (e.g. Screening invasive procedures)

A Serious Adverse Event (SAE) is any adverse event that meets any of the following criteria:

- Is Fatal
- Is Life threatening
- Requires of Prolongs inpatient hospitalization
- Is a congenital anomaly/birth defect in a neonate/infant born to a mother exposed to study drug/agent.
- Results in disability or permanent damage.

In this study all adverse events and Serious adverse events will be documented and assessed for treatment relation. The Radiation Therapy Oncology Group (RTOG) grading scale will be used to grade both acute and late toxicities; NCI CTCTAE 5.0 may be used for non-radiation related AEs. Pregnancy reporting will begin with first dose of LDRT through 6 months post-dose. The DSMC will be alerted in the event of an SAE and will meet within 24 hours. The adjudicating of the SAE causality will be performed jointly in consensus by the Principal Investigator and the DSMC.

# **STUDY POPULATION**

# **Inclusion criteria**

1. Be age ≥18 years
2. Have had a positive rtPCR test confirming the diagnosis of COVID-19 from nasopharyngeal swab, Sputum or lower respiratory tract sample
3. Have had clinical signs of severe acute respiratory syndrome or pneumonia (dyspnea, cough, with need for oxygen support at the time of enrolment)
4. Documented SpO2 < 94% while on ambient air during the screening window, and requires supplemental oxygen to maintain SpO2 ≥ 94% during the screening window.
5. Cohort 1: currently not on mechanical ventilation but would need mechanical ventilation if condition worsens (pre-Vent)
6. Cohort 2: currently on mechanical ventilation for ≤ 5 days (on-Vent)
7. Have visible consolidations/ground glass opacities on chest x-ray or computed tomography consisted with Covid 19 pneumonia.
8. The patient, according to the treating physician, has received medical treatment considered best practice as per the institution’s guidelines*, and does not show improvement of Covid pneumonia (i.e., inability to improve oxygenation despite current treatment, need to increase oxygenation and ventilatory support, and/or declining clinical status at current management)
9. Willingness and ability of the subject to comply with scheduled visits, protocol-specified laboratory tests, other study procedures, and study restrictions.
10. Evidence of a signed informed consent/assent indicating that the subject is aware of the infectious nature of the disease and has been informed of the procedures to be followed, potential risks and discomforts, potential benefits, and other pertinent aspects of study participation.

*institutional covid 19 treatment guidelines provided as Attachment

# **Exclusion Criteria**

1. Pre-existing lung comorbidity such as severe COPD, severe uncontrolled asthma or heart failure or concomitant active systemic infection.
2. Pre-existing dependency on supplemental oxygen prior to diagnosis of Covid 19 pneumonia
3. Patients on mechanical ventilation requiring 100% FIO2 and > 8mm PEEP to maintain SpO2 > 94% will not be eligible
4. Unwilling or unable to following protocol procedures
5. Pregnant and/or planned to be pregnant within in next 6 months
6. Hemodynamic and Ventilatory instability precluding transfer to Radiation Oncology department
7. Participation in another clinical trial involving an investigational product.

# **STUDY SITE**

# **Aga Khan University Hospital, Nairobi**

The study will be done at Aga Khan University (AKU) which is a private, not for profit, university and teaching hospital system that comprises 13 campuses, 7 hospitals and 217 outreach medical centers across 8 countries. The Aga Khan University Hospital, Nairobi (AKUH, N) provides tertiary and secondary level healthcare services and currently has a bed capacity of 300 and operates under the aegis of the Aga Khan Health Services.

The AKUHN approach to care is guided by core principles of Quality, Access, Impact and Relevance and its healthcare practices are of the highest quality, as benchmarked against international standards including accreditation by:

- - - Joint Commission International (JCI)
    - College of American Pathologists (CAP)
    - International Quality Assurance 9001:2008
    - South African National Accreditation Service (SANAS)

The division of infectious disease (ID), led by a team of highly trained and qualified specialists, who are nationally and internationally recognized for their work in a variety of ID sub-specialties most notably in research related to HIV medicine focusing on novel therapies, co-infection with other viruses such as HPV, the epidemiology and epigenetics of HIV, HIV drug resistance, and response to antiretroviral therapy (ART).

This team provides Infectious diseases coverage for both the wards and intensive care units and strives to ensure excellent patient care and patient safety. The division has a state-of-the-art microbiology laboratory with the capability to perform various specialized tests and contribute to the infection control program at the hospital and strongly believe and encourage best infection control practices to enhance patient care and safety. The division of Infectious diseases works closely with the section of Pulmonary and Critical Care Medicine and strives in ensuring excellent patient care and patient safety. Aga Khan University Hospital, Nairobi has a state-of-the-art facility that provides 24-hour monitoring to patients in the ICU and HDU run by our locally and internationally Critical Care Medicine trained faculty 24 hours a day, 7 days-a-week with expertise in invasive and noninvasive critical care medicine.

The Infectious disease and Critical care teams work in close relationship with our other sections, including the Departments of Radiology, Pathology, Nutrition and Laboratory, ensuring a multidisciplinary and expedited approach to patient care.

# **Geographical Location**

Aga Khan University Hospital is located in the Parklands area of Nairobi and serves a patient catchment area that includes Kenya, Somalia, South Sudan, Uganda, Rwanda, Democratic Republic of Congo, Tanzania, Ethiopia, Burundi, Djibouti, Nigeria. AKUHN has an 11-bed Intensive Care Unit (ICU) and a 16-bed High Dependency Unit (HDU). with up-to-date medical care including invasive monitoring (intracranial pressure and hemodynamic monitoring), organ support such as invasive and non-invasive mechanical ventilation and bedside renal replacement therapy (dialysis). In response to the COVID-19 pandemic, AKUHN has set up a 44-bed field hospital with an option to scale-up to 100-bed field-hospital. In addition, the hospital has made plans to double the number of patients that can be supported on artificial ventilation.

# **Language**

The patients served and managed at AKUH, N are primarily English- and Swahili-speaking. Both English and Swahili consent forms will be made available to the potential participants.

# **Radiotherapy (RT) facilities**

The Radiotherapy Unit is located at the basement floor of the Heart and Cancer Centre and is easily accessible for transfer of patients eligible to participate in this study. The Unit has two (2) linear accelerators that will be used to deliver the LDRT and is equipped with CCTV and audio systems that are both present for monitoring the patient while the treatment is on-going.  This includes the ability to visualize any equipment used for monitoring vital signs. Treatment planning software is available for dose calculations and portal imaging can be performed to verify that treatment has been delivered to the area of interest. Additionally, treatment will be permanently documented on the radiation treatment system (Varian). The unit has a fully stocked crash cart and an extra oxygen tank is available in the event of an emergency.

In light of the infectious nature of COVID 19 and to reduce risk of exposure the Radiotherapy unit has established the following procedures:

- 1. One Linear accelerator will be dedicated for treating only cancer patients.  The second machine will be used to treat COVID patients.  It will then revert back to treating cancer patients after complete cleaning and sterilization and downtime overnight. The Linac Brand is Varian model Dx, capable of delivering either 6Mv (preferred) or 15Mv photos. The variation will depend on patient separation (thickness). Equipment calibration is performed approximately every 6 months. Calibration for dose per monitor unit, beam stability, and output is performed daily. This information is maintained in the Department-specific files.
  2. Treatment planning will be done using diagnostic CT chest and/or CXR.  The CT simulator will be used as back up option for planning if necessary.
  3. By using information from diagnostic scans/x-rays the radiotherapy team will pre-plan the treatment prior to the patient’s arrival and thereby reduce the time spent in the department by the COVID patient
  4. Radiation is delivered when electric power is turned. At the time of delivery only the patient is permitted to be in the bunker. There is no active radiation source in the bunker, such that there is no accidental exposure. The technical staff are trained to ensure no one is left behind before turning on the beam. Those involved in delivering the treatment wear TLD badges (also as part of routine operations of the Department). Limited critical care staff will assist in patient transfer inside the bunker and will exit prior to “beam on”. See Section 4.4 Risk mitigation strategies for additional details.
  5. Radiation Protection Board of Kenya (KRPB) has routinely evaluated procedures and processes of the department including facility layout, emergency procedures, dose per monitor unit, beam stability and output, etc. License has been renewed annually and we continue to meet all standards for safe operation of a radiation facility.

# **The Radiotherapy staff.**

The RT unit is properly staffed with the following cadre of trained and experienced staff:

1. Qualified Radiation Therapy Technicians (5)
2. Certified medical physicist
3. Oncology Nurses (2) (supplemented by ward/ICU nurse)
4. Radiation Oncologists (3)
5. Hospital Trained cleaning crew
6. Full time engineer

# **Clinical Research Unit:**

The Aga Khan University Hospital, Nairobi has a Clinical Research Unit (CRU) with specific focus in conducting ICH-GCP/FDA compliant Clinical Research. The CRU is well-staffed by trained personnel with expertise in the following research related tasks:

- - - Clinical Research Nursing: Make CRF entries/resolve queries; Collect medical information; Vital signs; Collect adverse events; Administer IP; Collect lab samples; Participant recruitment and retention
    - Investigational Drug Services: Manage IP receipt/return; Prepare and dispense IP; Unblinding procedures
    - Regulatory Affairs: Maintain essential documents; Manage submissions to ethics and regulatory authorities
    - Contracts and Budgets: Maintain legal and financial documents; Ensure appropriate invoicing to the sponsor; Ensure appropriate processes for research covered procedures versus patients’ standard of care
    - Data Management: Make CRF entries/resolve queries; Maintain the patients’ research documents
    - Clinical Research operations: Oversight of AKU’s Clinical Research Unit’s studies and staff; support all research staff with their tasks
    - Investigator: Obtain Informed Consent; Assess eligibility; Make CRF entries/resolve Queries; Medical decisions/review; Collect medical information; Vital signs; Assess Ordinal Scale; Collect/Assess Adverse Events; Administer IP; Conduct Physical Exam; Collect Lab Samples; Maintain Essential Documents; IP processes

# **BEST PRACTICE MANAGEMENT OF COVID 19 PNEUMONIA**

This study looks to investigate the safety and feasibility of low dose whole-lung radiation therapy (LDRT) in critically-ill patients with COVID-19 pneumonia.

AKUHN has best practice management guidelines for Covid 19 pneumonia (*Appendix ii).* All patients at AKUHN are treated based upon their clinical signs and symptoms and in accordance to these guidelines. Patients enrolled on this study can have received any or all of the medical treatment (e.g. dexamethasone, remdesivir, convalescent plasma, tocilizumab) outlined in the best practice management guidelines for Covid 19 pneumonia.

# **Management of COVID-19 at AKUH, N**

Approximately 60% of COVID-19 patients have not required mechanical ventilation and approximately 26% of COVID-19 patients have required management in the Intensive Care Unit. The diagnosis of COVID-19 pneumonia at AKUH, N is confirmed when a patient with fever or respiratory symptoms such as cough, difficulty in breathing, sore throat or desaturation on room air has a positive SARS-CoV-2 PCR from a nasopharyngeal swab or lower respiratory tract specimen.

Patients are then classified according to the severity of their symptoms

- asymptomatic
- mild (fever, cough, sore throat with no difficulty in breathing or desaturation)
- moderate disease (difficulty in breathing but saturation at 94% or more on room air at rest)
- severe disease (respiratory rate, ≥30 breaths per minute, oxygen saturation ≤93%; ratio of partial pressure of arterial oxygen to fraction of inspired oxygen <300 and lung infiltrates >50% of the lung field involved)
- critical disease (mechanical ventilation required, multiple organ dysfunction).

Management of COVID-19 is based on the severity scale. Patients with asymptomatic or mild disease are offered home isolation and follow up according to the current MoH guidelines. Those unable to fulfil criteria for home admission are admitted for observation and symptomatic management with analgesics and antipyretics.

Those with moderate disease are hospitalized. Oxygen is given if saturation falls beneath 94%. Monitoring with laboratory investigations is done if and when required. Laboratory parameters that may be requested include inflammatory markers (LDH, ferritin, D-dimers, CRP, procalcitonin), organ function tests (liver function, renal function, troponin-T), and evaluation of oxygenation status (arterial blood gas). No specific therapy is offered other than supportive measures with oxygen, prophylactic anticoagulation, and fever management. Self proning is taught and encouraged. Anticipated complication such as cytokine storms, specific organ dysfunction, or thrombotic episodes are managed in conjunction with the relevant medical specialist.

Those with severe and critical disease are managed in the Intensive Care Unit in conjunction with the critical care team. Those with severe disease are offered high flow oxygen with face masks, non-rebreather masks and high flow nasal cannula when available. Monitoring is round the clock in case mechanical ventilation is required. The same laboratory monitoring as is indicated for moderate disease is carried out. Self proning is also done at this point. Management is supportive. Those requiring mechanical ventilation and intubation are managed with the ARDS protocol that has been previously in place. Organ dysfunction is managed in conjunction with specialists such as nephrologists and cardiologists. Other therapies that may be offered include full anticoagulation where thrombotic disease is suspected on the basis of rising D-dimer levels and worsening hypoxemia.

Discharge of the patients is based on resolution of hypoxaemia with ability to saturate at 94% or above on room air. Previously, two negative nasopharyngeal swabs have been required, but this is under review given the current MoH guidelines on home based isolation.

Medical management of patients at AKUHN is determined by a team of consultants, which include infectious disease, pulmonology, intensive care, hematology and others as needed. Treatment depends on clinical condition and severity of symptoms. Guidelines have recently been developed in order to standardize medical management of these patients (see Appendix ii).

# **RECRUITMENT AND INFORMED CONSENT**

The study will recruit patients hospitalized at AKUHN who meet eligibility criteria and can provide consent.

The study will recruit up to twenty **(20)** patients hospitalized at AKUHN who meet eligibility criteria and can provide consent. We intend to pre-screen approximately twenty-five (**25**) to **e**nrol the target number of patients.

The study will enrol 2 cohorts of patients, those that are on maximum oxygen support and are at risk of needing mechanical ventilation (Cohort 1: pre-Vent) and those who are already on mechanical ventilation (Cohort 2: on-Vent). Enrolment of each cohort will be done simultaneously and independently. Patients in Cohort 1 will likely be able to provide consent on their own. Patients on mechanical ventilation will need next of kin to provide consent. Procedures for both have been outlined and we have experience with the processes in the context of infection control guidelines for Covid19.

Eligible study participants (or next of kin) will be informed about the trial through an informed consent process conducted by the study investigators. Patients (or next of kin) will then be provided with the informed consent form and given time to review the information therein, ask questions and seek clarification before providing consent.

Institutional infection control policies for COVID-19 and similar highly transmissible and infectious viruses requires that no content should be taken out from a COVID-19 patient’s room except for the purpose of incineration and disinfection. For patients providing informed consent, the following process will be used to ensure the compliance with the ICF process:

- Consent information will be presented to patient by a study team member in the presence of a witness who is not a study team member.
- If patient agrees to participate in the study, he/she will sign and date the consent form.
- The investigator will take a photograph of the signed consent document.
- Photo image of the signed signature page of the consent form will be printed.
- The witness together with the person administering consent will sign and date the printed ICF.
- This will be placed in the subject’s chart.
- A printed copy of the fully signed ICF will be given to the participant or emailed to them.
- The Investigator will make a note in the patient’s medical record documenting the patient’s consent to participate in the study.

In the event that the patient is too critically ill to provide consent, the next of kin/ legally authorized representative will be provided with the consent information in the presence of a witness who is not a study team member and both will sign and date the ICF. A copy of the fully signed ICF will be given to the legal representative.

In cases where the legally authorized representative or next of kin gives consent, the investigators will conduct and informed consent discussion with the participant and once the he/she regains capacity during the course the study and written consent to participate will be obtained from the patient.

Upon consenting participants will undergo the study related screening procedures outlined in Table 1. “Schedule of events” prior to administration of the LDRT. The administration of the LDRT will only be done on participants who meet the screening inclusion criteria.

Additionally, the study participants and their next of kin, if applicable, will be given counselling on radiotherapy, including what it entails, the different types of radiotherapy and what to expect during radiotherapy, possible side effects and dietary specifications (if indicated). Psychosocial counselling for patients and next of kin is also provided to all Covid 19 patients as part of institutional guidelines.

In the event that IERC approved amendments of study procedures are made to this protocol, the informed consent form will be revised to reflect these amendments to study procedures and the participants will be approached to provide new consent (re-consented)

**The participants will have the right to continue on the study or withdraw from the study.**

Participants who are not able to communicate and comprehend the English or Swahili informed consent will not be enrolled on this study.

# **STUDY PROCEDURES**

# Screening

Procedures performed per standard of care prior to consent may be used for screening purposes if they are within 7 days prior to Day 1 (treatment):

- Informed consent
- Medical history, including current medications
- Demographics
- Ordinal score
- Vital Signs and Oxygenation
- Physical exam
- Routine labs
- Pre-dose radiograph (Chest CT/X-Ray)
- Special labs (stored)

Upon confirmation of eligibility, the following procedures will be performed on days indicated below throughout the participant’s hospital stay. If the participant is discharged prior to Day 28, the Discharge procedures will be conducted:

# Days 1, 3, 7, 14, and 28:

- - Ordinal Score
  - Vital Signs and Oxygenation
  - Physical exam
  - Routine labs
  - Special labs (stored)
  - Day 1 only: LDRT 100 cGy (single dose)
  - Day 1 only: Safety monitoring for 4 hours after receiving LDRT
  - Day 14 only: For participants discharged prior to Day 14, a follow-up visit/phone call for safety (adverse events and concomitant medications) will be conducted.
  - Adverse Events
  - Concomitant Medications

# Days 2, 4-6:

- - Day 2 only: Ordinal Score
  - Vital Signs and Oxygenation
  - Adverse Events
  - Concomitant Medications

# Discharge

If participant’s hospital stay is less than 28 days. If participant is discharged prior to Day 14, then monitoring for safety (Adverse Events, Concomitant Medications) will continue through Day 14:

- - Ordinal Score
  - Vital Signs and Oxygenation
  - Physical exam
  - Routine labs
  - Special labs (stored)
  - Pre-dose radiograph (Chest CT/X-Ray), per investigator discretion using the same modality as Screening, if possible
  - Adverse Events
  - Concomitant Medications

**Specific details on procedures are as follows:**

1. **Ordinal Score (on a scale of 1-7) will be determined according to the following criteria:**

1: Discharged (or “ready for discharge” as evidenced by normal body temperature and respiratory rate, and stable oxygen saturation on ambient air or ≤2L supplemental oxygen)

- Includes patients pending discharge due to non-medical or administrative reasons

2: Non-ICU hospital ward (or “ready for hospital ward”), not requiring supplemental oxygen

- - Includes patients in ICU for administrative/non-medical reasons, who don’t require ICU setting and supplemental oxygen

3: Non-ICU hospital ward (or “ready for hospital ward”), requiring supplemental oxygen

- - Includes patients in ICU for administrative/non-medical reasons, who don’t require ICU setting, but require supplemental oxygen

4: ICU or non-ICU hospital ward, requiring non-invasive ventilation or high-flow oxygen

- - Includes patients requiring ICU care, but waiting for ICU setting
  - Includes patients in ICU for administrative/non-medical reasons, who don’t require ICU setting, but require non-invasive ventilation or high-flow oxygen

5: ICU, requiring intubation and mechanical ventilation

6: ICU, requiring extracorporeal membrane oxygenation (ECMO) or mechanical ventilation and additional organ support (e.g., vasopressors, renal replacement therapy)

7: Death

1. **Vital Signs and Oxygenation will be collected as follows:**

- Temperature, blood pressure, respiratory rate, pulse oximeter, oxygenation %, and setting (amount, type)
- To be collected as clinically indicated (due to AEs), in addition to the required time points

1. **Routine labs are as follows:**

- CBC+ diff: WBC count, RBC count, hemoglobin, hematocrit, platelet count, differential count
- Lytes: Bicarbonate, sodium, potassium, chloride, BUN creatinine
- LFT: Total bilirubin, ALP, ALT, and AST
- Ferritin
- CRP
- D-dimers
- Screening only: Pregnancy test for women of childbearing potential

1. **Special labs (stored) are as follows:**

A blood sample of 20ml will be collected and stored for future analysis (i.e., immune profile and cytokine measurements: IL-2, IL-7, granulocyte colony stimulating factor, interferon-γ inducible protein 10, monocyte chemoattractant protein 1, macrophage inflammatory protein 1-α, and tumour necrosis factor-α, ferritin, IL-6, IL-10, IFN- α, IFN- γ).

1. **Radiation Process:**

- Patient positioning and simulation: In order to minimize risk of cross infection we will use diagnostic images that will have been performed as part of routine care of patient. We will not be simulating the patient in our department, but a portal image will be taken just prior to treatment delivery, where the parameters from the diagnostic imaging are input into the treatment planning system to ensure accuracy of calculations, appropriate lung coverage, and safety of the treatment.
- Patient Education:
  - On the treatment day if the patient is conscious, an explanation of the treatment procedure is provided, side effects reviewed and consent ensured. Otherwise, the procedure is explained to next of kin.
  - Documentation of patient education and any images taken will be added in the notes section of the patient’s chart.
- Preparation guidelines
  - Patient is identified correctly; the treating staff introduces themselves to the patient.
  - The patient should remove all clothing from the waist up, change into a gown/shirt and remove shoes for treatment.
  - Before setting up the patient, the couch top and any other accessory is covered with a sheet.
  - Patient is then positioned as per setup instructions.
- Following radiotherapy administration, patient will be monitored to ensure he/she is safe to be transported back to the hospital ward. Safety monitoring will continue through 4 hours post-treatment, and routine monitoring will resume per standard of care.

# **DATA MANAGEMENT**

The investigator team will monitor data frequently for quality and completeness. Data from source documents will be transferred into a case report forms (CRFs). All CRFs will be kept under lock and key, maintained by the Principal Investigator. Only authorized personnel (investigator and CRU study team) will have access to the participant study documentation.

The AKUH. N Clinical Research Unit (CRU) will be responsible for risk based monitoring. The following source data verification will be assessed as follows for all participants:

1. Eligibility check,
2. Informed consent process
3. Serious adverse events monitoring

In addition, ten percent of all participant charts will be randomly selected and assessed for 100% of source data verification and compliance with International Conference on Harmonization Good Clinical Practice (ICH-GCP). The Internal audit of the participant charts will be done by the Clinical Research Unit Quality Assurance Personnel, who will not be part of the study team. If significant issues are identified during the above noted review, additional participant charts will be selected for review to ensure protocol adherence, at the discretion of the CRU staff.

# **CONFIDENTIALITY**

All study documentation containing personal information relating to study subjects like consent forms and other documents that might link subject ID with other subject personal details will be kept in a secure locked area with limited access at Aga Khan University Hospital. Such documentation will only be made available to authorized personnel. These study documents will be made available to the investigators, clinical personnel who require this information to treat the patient and the above-mentioned personnel for inspection or auditing reasons. All electronic data kept at the investigator’s site will be kept secure. Computer access will only be made available to authorized personnel.

# **SAMPLE SIZE**

This is a pilot Phase 1b/2, proof of principle, study to determine safety and feasibility of a single dose LDRT for the treatment of severe Covid 19 pneumonia patients who have nearly exhausted conventional available options. Sample size is based on having sufficient patients to reasonably provide descriptive data for feasibility, toxicity and indication of efficacy:

- Cohort 1 (pre-Vent): 5 patients will be treated. If acute treatment related SAE is observed in ≤ 1 of 5 patients at 24h following LDRT, then 5 additional patients will be treated.
- Cohort 2 (on-Vent): 5 patients will be treated. If acute treatment related SAE is observed in ≤ 1 of 5 patients at 24h following LDRT, then 5 additional patients will be treated.

# **STATISTICAL ANALYSIS**

Descriptive statistics will be used to determine proportion of patients experiencing toxicity, improvement/worsening of oxygenation status, weaning off oxygenation or ventilation, discharge or death on/before 14d and on/before 28d. All endpoints will be summarized descriptively, using mean, median, interquartile range, minimum/maximum, and standard deviation for continuous endpoints, and frequency and percentage for categorical endpoints. The data from the 2 cohorts will be analysed independently.

# **ETHICS**

# **Study Conduct**

The trial will be conducted in compliance with the principles and guidelines of the Declaration of Helsinki (Version 2013), and to principles of Good Clinical Practice, Institutional Ethics Review Committee and regulations in Kenya.

# **Ethics Approval**

This protocol and supporting documentation will be submitted to Aga Khan University, Nairobi Institutional Ethics Review Committee for review and approval and to the Poisons and Pharmacy board (PPB) for review and approval. Implementation of the study will not occur until approvals from both entities are received. In the event the study shows benefit, the study team will seek approval from the AKUHN IERC and DSMC, the study could be expanded with the approval of the IERC to patients from outlying hospital could potential be transferred for a one-time treatment at the AKUHN RT unit. In addition, AKUHN investigator will train and assist colleagues at other RT units in Kenya to adopt this protocol in consultation with the regulatory authorities.

A research license will be sought from the National Council of Science, Technology and Innovation (NACOSTI).

Ethical approval from the IERC will be sought in separate amendment or protocol for the future analysis - for research purposes - of special labs collected.

# **Protocol amendments**

All amendments and modifications will be submitted to Aga Khan University, Nairobi Ethics Review Committee and Pharmacy and Poisons board for review and approval. No changes in protocol conduct will be implemented until approval is obtained from the Aga Khan University, Nairobi Ethics Review Committee and Pharmacy and Poisons board, unless required to eliminate apparent immediate hazards to the study participants.

# **Protocol deviations**

- Major Protocol violations and protocol deviations shall be reported to AKU, N IERC within 48 hours of the PI becoming aware of them
- Minor Protocol deviations will be reported to AKU, N IERC through a cumulative log with the annual continuing review report
- Major Protocol violations and protocol deviations shall be reported to the PPB within 7 days of the PI becoming aware of them.
- Minor Protocol deviations will be reported to PPB through a cumulative log with the annual continuing review report
- All deviations will also be summarized in the continuing review reports that are submitted AKU, N IERC and PPB
- Any suspensions (to include continuing review lapses), clinical holds (voluntary or involuntary), or terminations of this research by an IRB, the institution, the Sponsor, or regulatory agencies will be promptly reported to AKU, N IERC and PPB

# **Serious adverse events reporting**

SAEs and SUSARs will be reported as soon as possible, but within 48 hours of the site becoming aware of the event to AKUN, IERC.

SAEs and SUSARs will be reported as soon as possible, but within seven days of the site becoming aware of the event to PPB.

Follow up reports will be submitted as soon as available.

# **Unanticipated events**

All non-serious unanticipated problems (events not involving risk to participants or others) will be reported in the continuing review report to AKUN, IERC and PPB.

All serious unanticipated problems involving risk to participants or others should promptly (within 48 hours) to AKUN, IERC and to PPB within 7 days.

# **Continuing Review Reports**

The PI will be responsible for submitting the required continuing review report and supporting documentation to the AKUN, IERC and PPB, allowing sufficient time for review and continuation determination prior to the established continuing review date. A closeout report will be submitted at the end of the approved study duration, or upon completion of the study activities, whichever comes first.

# **Inspections and Audits**

Knowledge of any pending compliance inspections/visits by government agency concerning clinical investigation or research, the issuance of Inspection Reports, warning letters or actions taken by any Regulatory Agencies including legal or medical actions and any instances of serious or continuing noncompliance with the regulations or requirements will be reported immediately to AKUN, IERC

# **Data Safety Monitoring Committee (DSMC)**

An independent Data Safety Monitoring Committee (DSMC) will be established and will provide oversight for the conduct of this study. The DSMC will function independently to specifically determine SAE and relationship to study treatment at 24h following the treatment of each of the first 5 patients in Cohort 1. The DSMC will review pertinent aspects of the study to assess subject safety, compliance with the protocol, data collection, and risk-benefit ratio. Following the monitoring review, the chair will provide approval for the study to continue or be halted.

Study staff will get approval by the DSMC for opening the second cohort if in the opinion of the DSMC ≤ 1 of the 5 patients in cohort 1 have experience a treatment related SAE within 24h of treatment.

Additionally, the DSMC will be alerted in the event of any SAE and will meet within 24 hours. The adjudication of the SAE causality will be performed jointly in consensus by the PI and DSMC.

# **STUDY RELATED INJURY**

Upon receipt of the IERC Approval, this study will be added to the AKU insurance/indemnity schedule of clinical trials, to cover the cost of injury resulting from their participation in this study.

# **COMPENSATION**

Participants will not be paid for taking part in this study. However, participants will each receive a self-care package (masks, gloves, and 50ml bottle of sanitizer) at the time of discharge from the hospital,

# **DISSEMINATION OF RESULTS**

A clinical study report containing the results of this trial will be made available to anyone who requests a copy; it will not include any patient-identifiable information.

A description of this clinical trial will be available on the Kenya Pharmacy and Poison’s Board clinical trial registry as required by Kenyan law, and with the Pan African Clinical Trials Registry.

# **POST TRIAL ACCESS**

In the event the study shows benefit, in consultation with regulatory authorities, an approximate cost package will be developed at AKUHN for treatment of Covid-19 pneumonia. In addition, AKUHN investigators will train and assist colleagues at other RT units in Kenya to adopt this treatment. The protocol used at AKUHN will be shared with the respective investigators.

# **BUDGET**

LDRT will be the only aspect of medical management that will experimental and beyond best practice of Covid 19 management. The cost for LDRT will be borne by the Department of Haematology- Oncology. The cost for PPE to be used for transportation to and from the RT unit and for the RT personnel will be borne by AKUHN.
“Special labs” will be drawn, processed and stored for future analysis and not bill to the patient. Patients will not be billed for any expenses related to the experimental treatment; this includes treatment expenses for adverse events that are related to the experimental treatment. All other charges are part of routine care of Covid 19 patients and will be billed to 3rd party or the patient, accordingly.

| **Item** | **Unit Cost Ksh** |
| --- | --- |
| PPE used during LDRT* | Ksh6,800 |
| Routine standard of are labs | Ksh24,100 |
| Urine pregnancy test* | Ksh1,500 |
| Special labs* | Ksh2,000 |
| Radiotherapy* | Ksh9,400 |

| **Personnel** | **Total Study Cost (Ksh)** |
| --- | --- |
| Data Management* | Ksh11,000 |
| Nurse* | Ksh33,188 |
| Investigator* | Ksh117,000 |
| Regulatory/monitoring* | Ksh69,750 |

| **Regulatory Items** | **Unit Cost Ksh** |
| --- | --- |
| PPB (one-time)* | Ksh100,000 |
| NACOSTI (annual)* | Ksh20,000 |

*the cost of these study related tests will be borne by the institution.

# **REFERENCES**

1. *Huang C, Wang Y, Li X, et al: Clinical features of patients infected with 2019 novel coronavirus in Wuhan, China. Lancet 395:497-506, 2020*
2. *Bhatraju PK, Ghassemieh BJ, Nichols M, et al: Covid-19 in Critically Ill Patients in the Seattle Region - Case Series. N Engl J Med, 2020*
3. *Calabrese EJ, Dhawan G: How radiotherapy was historically used to treat pneumonia: could it be useful today? Yale J Biol Med 86:555-70, 2013*
4. *Calabrese EJ, Dhawan G: The historical use of radiotherapy in the treatment of sinus infections. Dose Response 11:469-79, 2013*
5. *Calabrese EJ: X-Ray treatment of carbuncles and furuncles (boils): a historical assessment. Hum Exp Toxicol 32:817-27, 2013*
6. *Dodds DR: Antibiotic resistance: A current epilogue. Biochem Pharmacol 134:139-146, 2017*
7. *Subbaraman N: Coronavirus tests: researchers chase new diagnostics to fight the pandemic. Nature, 2020*
8. *Fauci AS, Lane HC, Redfield RR: Covid-19 - Navigating the Uncharted. N Engl J Med, 2020*
9. *Wang D, Hu B, Hu C, et al: Clinical Characteristics of 138 Hospitalized Patients With 2019 Novel Coronavirus-Infected Pneumonia in Wuhan, China. JAMA, 2020*
10. *Bai Y, Yao L, Wei T, et al: Presumed Asymptomatic Carrier Transmission of COVID-19. JAMA, 2020*
11. *Ai T, Yang Z, Hou H, et al: Correlation of Chest CT and RT-PCR Testing in Coronavirus Disease 2019 (COVID-19) in China: A Report of 1014 Cases. Radiology:200642, 2020*
12. *J.H. Beigel, K.M. Tomashek, L.E. Dodd, et Remdesivir for the Treatment of Covid-19 — Preliminary Report, DOI: 10.1056/NEJMoa2007764*
13. *Giovanni Guaraldi*, Marianna Meschiari* et al, Tocilizumab in patients with severe COVID-19: a retrospective cohort study, Tociluzumab – the Lancet, doi.org/10.1016/ S2665-9913(20)30173-9*
14. *Peter Horby, F.R.C.P., Wei Shen Lim, F.R.C.P., Dexamethasone in Hospitalized Patients with Covid-19 — Preliminary Report - DOI: 10.1056/NEJMoa2021436*
15. *Amy Dockser Marcus, Study Points to Efficacy of Convalescent Plasma for Covid-19, The Wall Street Journal, -* [*https://www.wsj.com/articles/study-points-to-efficacy-of-convalescent-plasma-for-covid-19-11590177304*](https://www.wsj.com/articles/study-points-to-efficacy-of-convalescent-plasma-for-covid-19-11590177304)
16. *Mehta P, McAuley DF, Brown M, Sanchez E, Tattersall RS, Manson JJ. COVID-19: Consider cytokine storm syndromes and immunosuppression. The Lancet (British edition). 2020;395(10229):1033-1034.*[*https://search.datacite.org/works/10.1016/s0140-6736(20)30628-0*](https://search.datacite.org/works/10.1016/s0140-6736(20)30628-0)*. doi: 10.1016/s0140-6736(20)30628-0.*
17. *James Conca Preliminary Data Suggests Low-Dose Radiation May Be Successful Treatment For Severe Covid-19* [*https://www.forbes.com/sites/jamesconca/2020/06/12/1st-human-trial-successfully-treated-covid-19-using-low-doses-of-radiation/#7ee0582adc69*](https://www.forbes.com/sites/jamesconca/2020/06/12/1st-human-trial-successfully-treated-covid-19-using-low-doses-of-radiation/#7ee0582adc69) *(this was confirmed by PI – peer reviewed publication is pending)*
18. *Montero A, Arenas M, Algara M. Low-dose radiation therapy: could it be a game-changer for COVID-19? [published online ahead of print, 2020 May 25]. Clin Transl Oncol. 2020;1-4. doi:10.1007/s12094-020-02401-y1.*
19. *Coperchini F, Chiovato L, Croce L, Magri F, Rotondi M. The cytokine storm in COVID-19: An overview of the involvement of the chemokine/chemokine-receptor system. Cytokine Growth Factor Rev. 2020;53:25-32.*[*http://www.sciencedirect.com/science/article/pii/S1359610120300927*](http://www.sciencedirect.com/science/article/pii/S1359610120300927)*. doi:*[*https://doi.org/10.1016/j.cytogfr.2020.05.003*](https://doi.org/10.1016/j.cytogfr.2020.05.003)*.*
20. *Rousseau JP, Johnson WM, GT H: The Value of Roentgen Therapy in Pneumonia Which Fails to Responde to the Sulfonamides. Radiology 38:281-9, 1942*
21. *Mehta P, McAuley DF, Brown M, et al: COVID-19: consider cytokine storm syndromes and immunosuppression. Lancet 395:1033-1034, 2020*
22. *Bracaglia C, Prencipe G, De Benedetti F: Macrophage Activation Syndrome: different mechanisms leading to a one clinical syndrome. Pediatr Rheumatol Online J 15:5, 2017*
23. *Grom AA, Horne A, De Benedetti F: Macrophage activation syndrome in the era of biologic therapy. Nat Rev Rheumatol 12:259-68, 2016*
24. *Jordan MB, Hildeman D, Kappler J, et al: An animal model of hemophagocytic lymphohistiocytosis (HLH): CD8+ T cells and interferon gamma are essential for the disorder. Blood 104:735-43, 2004*
25. *Morimoto A, Nakazawa Y, Ishii E: Hemophagocytic lymphohistiocytosis: Pathogenesis, diagnosis, and management. Pediatr Int 58:817-25, 2016*
26. *Marquez SD, Lum BL, McDougall IR, et al: Long-term results of irradiation for patients with progressive Graves' ophthalmopathy. Int J Radiat Oncol Biol Phys 51:766-74, 2001*
27. *Wakelkamp IM, Tan H, Saeed P, et al: Orbital irradiation for Graves' ophthalmopathy: Is it safe? A long-term follow-up study. Ophthalmology 111:1557-62, 2004*
28. *Betz N, Ott OJ, Adamietz B, et al: Radiotherapy in early-stage Dupuytren's contracture. Long-term results after 13 years. Strahlenther Onkol 186:82-90, 2010*
29. *Heyd R, Dorn AP, Herkstroter M, et al: Radiation therapy for early stages of morbus Ledderhose. Strahlenther Onkol 186:24-29, 2010*
30. *Incrocci L, Wijnmaalen A, Slob AK, et al: Low-dose radiotherapy in 179 patients with Peyronie's disease: treatment outcome and current sexual functioning. Int J Radiat Oncol Biol Phys 47:1353-6, 2000*
31. *Niewald M, Wenzlawowicz KV, Fleckenstein J, et al: Results of radiotherapy for Peyronie's disease. Int J Radiat Oncol Biol Phys 64:258-62, 2006*
32. *Schuster J, Saraiya S, Tennyson N, et al: Patient-reported outcomes after electron radiation treatment for early-stage palmar and plantar fibromatosis. Pract Radiat Oncol 5:e651-8, 2015*
33. *Seegenschmiedt MH, Attassi M: [Radiation therapy for Morbus Ledderhose -- indication and clinical results]. Strahlenther Onkol 179:847-53, 2003*
34. *Hanley B, Lucas SB, Youd E, et al: Autopsy in suspected COVID-19 cases. J Clin Pathol, 2020*
35. *Shi S, Qin M, Shen B, et al: Association of Cardiac Injury With Mortality in Hospitalized Patients With COVID-19 in Wuhan, China. JAMA Cardiol, 2020*
36. *Pollom EL, Chin AL, Diehn M, et al: Normal Tissue Constraints for Abdominal and Thoracic Stereotactic Body Radiotherapy. Semin Radiat Oncol 27:197-208, 2017*
37. *Zhao J, Yorke ED, Li L, et al: Simple Factors Associated With Radiation-Induced Lung Toxicity After Stereotactic Body Radiation Therapy of the Thorax: A Pooled Analysis of 88 Studies. Int J Radiat Oncol Biol Phys 95:1357-1366, 2016*
38. *Suzuki G, Ogata T, Aibe N, et al: Effective heart-sparing whole lung irradiation using volumetric modulated arc therapy: a case report. J Med Case Rep 13:277, 2019*
39. *Wong JYC, Filippi AR, Dabaja BS, et al: Total Body Irradiation: Guidelines from the International Lymphoma Radiation Oncology Group (ILROG). Int J Radiat Oncol Biol Phys 101:521-529, 2018*
40. *Topkan E, Yildirim BA, Guler OC, et al: Safety and palliative efficacy of single-dose 8-Gy reirradiation for painful local failure in patients with stage IV non-small cell lung cancer previously treated with radical chemoradiation therapy. Int J Radiat Oncol Biol Phys 91:774-80, 2015*
41. *Videtic GM, Paulus R, Singh AK, et al: Long-term Follow-up on NRG Oncology RTOG 0915 (NCCTG N0927): A Randomized Phase 2 Study Comparing 2 Stereotactic Body Radiation Therapy Schedules for Medically Inoperable Patients With Stage I Peripheral Non-Small Cell Lung Cancer. Int J Radiat Oncol Biol Phys 103:1077-1084, 2019*
42. *Guo T, Fan Y, Chen M, et al: Cardiovascular Implications of Fatal Outcomes of Patients With Coronavirus Disease 2019 (COVID-19). JAMA Cardiol, 2020*
43. *Bonow RO, Fonarow GC, O'Gara PT, et al: Association of Coronavirus Disease 2019 (COVID-19) With Myocardial Injury and Mortality. JAMA Cardiol, 2020*
44. *Russell AH, Clyde C, Wasserman TH, et al: Accelerated hyperfractionated hepatic irradiation in the management of patients with liver metastases: results of the RTOG dose escalating protocol. Int J Radiat Oncol Biol Phys 27:117-23, 1993*
45. *Yang X, Yu Y, Xu J, et al: Clinical course and outcomes of critically ill patients with SARS-CoV-2 pneumonia in Wuhan, China: a single-centered, retrospective, observational study. Lancet Respir Med, 2020*
46. *Joseph N, McWilliam A, Kennedy J, et al: Post-treatment lymphocytopaenia, integral body dose and overall survival in lung cancer patients treated with radical radiotherapy. Radiother Oncol 135:115-119, 2019*
47. *Prummel MF, Mourits MP, Blank L, et al: Randomized double-blind trial of prednisone versus radiotherapy in Graves' ophthalmopathy. Lancet 342:949-54, 1993*
48. *Seegenschmiedt MH, Keilholz L, Gusek-Schneider G, et al: [Endocrine orbitopathy: comparison of the long-term result and classification after radiotherapy]. Strahlenther Onkol 174:449-56, 1998*
49. *Kahaly GJ, Rosler HP, Pitz S, et al: Low- versus high-dose radiotherapy for Graves' ophthalmopathy: a randomized, single blind trial. J Clin Endocrinol Metab 85:102-8, 2000*
50. *Nakamura N, Kusunoki Y, Akiyama M: Radiosensitivity of CD4 or CD8 positive human T-lymphocytes by an in vitro colony formation assay. Radiat Res 123:224-7, 1990*
51. *Yovino S, Kleinberg L, Grossman SA, et al: The etiology of treatment-related lymphopenia in patients with malignant gliomas: modeling radiation dose to circulating lymphocytes explains clinical observations and suggests methods of modifying the impact of radiation on immune cells. Cancer Invest 31:140-4, 2013*
52. *Paun A, Kunwar A, Haston CK: Acute adaptive immune response correlates with late radiation-induced pulmonary fibrosis in mice. Radiat Oncol 10:45, 2015*
53. *Zheng X, Guo Y, Wang L, et al: Recovery Profiles of T-Cell Subsets Following Low-Dose Total Body Irradiation and Improvement With Cinnamon. Int J Radiat Oncol Biol Phys 93:1118-26, 2015*
54. *Olschewski T, Seegenschmiedt MH: Radiotherapy for bony manifestations of Langerhans cell histiocytosis. Review and proposal for an international registry. Strahlenther Onkol 182:72-9, 2006*
55. *Laird J, Ma J, Chau K, et al: Outcome After Radiation Therapy for Langerhans Cell Histiocytosis Is Dependent on Site of Involvement. Int J Radiat Oncol Biol Phys 100:670-678, 2018*
56. *Classen CF, Minkov M, Lehrnbecher T: The Non-Langerhans Cell Histiocytoses (Rare Histiocytoses) - Clinical Aspects and Therapeutic Approaches. Klin Padiatr 228:294-306, 2016*

# **APPENDICES**

1. Informed consent form (English)
2. Informed Consent form (Swahili)
3. AKUH, N Inpatient COVID 19 management protocol
4. Case Report Form

# ***Appendix (i)*** *Informed Consent Form (English Version)*

| **Project Information** | |
| --- | --- |
| Project Title: | A pilot phase Ib/II study of whole-lung low dose radiation therapy (LDRT) in the treatment of severe Covid 19 pneumonia patients on (or requiring) mechanical ventilatory support. |
| Principal Investigator | Prof. Mansoor Saleh |
| Organization | Aga Khan University Hospital, Nairobi |
| Sponsor | Aga Khan University Hospital, Nairobi |

1. **INTRODUCTION**

My name is Mansoor Saleh in the department of Hematology-Oncology at the Aga Khan University Hospital Nairobi. Together with my colleagues, we are doing a clinical research trial on the use of low dose radiation therapy in the treatment of severely ill COVID-19 patients. We will be treating 2 separate groups (cohorts) of patients. Cohort 1 will be patients whose oxygen level is not improving and the patient may need to be placed on a ventilator (machine that provides oxygen into the patient’s lungs and helps them breath) if things worsen. Cohort 2 will be those patients who are already on a ventilator for no more than 5 days.

COVID-19 is a worldwide pandemic caused by the novel coronavirus. It causes fever, cough and fatigue. While most patients seem to recover, some patients get severe complications often involving the lungs and are unable to breath and need to receive high level of oxygen or be place on a ventilator to enable them to survive. While there are a number of treatments that are being tried for patients with Covid pneumonia, there are many patients who have exhausted these options or unable to receive them. Even under the best of circumstances, a small number of patients get severely ill and are unable to get enough oxygen to sustain their body function. Under those circumstances, patients may need to receive mechanical ventilation through a machine that will provide oxygen and assist the patient’s own breathing.

The treatment being offered is a single dose of radiation to the lung, which is where the virus is causing the most damage and which is why patients are unable to breath and get enough oxygen into their body. It appears that the patient’s immune system, which his supposed to fight the virus has become overactive and producing substances that cause flooding of the lung tissue whereby immune cells and fluids block free flow of oxygen from the lung into the blood system. This overactive immune system and the flooding of the lung tissue is terms cytokine storm (cytokines are those substances produced by the immune system during the fight against the virus). This cytokine storm results in what we call ARDS (acute respiratory distress syndrome), which is the flooding of the lung tissue with immune cells and fluid that causes the lung tissue to become scarred and unable to transfer oxygen from the air into the blood system. On the chest x-ray or a CT scan, ARDS is seen as a “dense clouding of the lung”.

Before the use of antibiotics to treat pneumonia, radiation was often used as one of the treatment for helping patients with viral pneumonia. Low doses of radiation have also been used to treat some diseases that result from over activity of the immune system. It is possible that this same strategy of low dose radiation can be used to treat the pneumonia, cytokine storm and ARDS (severe lung inflammation) caused by COVID-19. Low dose radiation to the lungs is supposed to be effective in reducing the inflammation caused by the virus and can help improve outcomes in critically ill patients.

The approving authorities for this study are:

- Aga Khan University Institutional Ethics Review Committee (IERC)
- Pharmacy and Poisons Board (PPB)
- National Council of Science, Technology and Innovation (NACOSTI)

1. **PURPOSE OF THIS RESEARCH STUDY**
   - You are being asked to participate in a research study designed to find out if low dose radiation therapy can be safely administered to patient with severe Covid 19 pneumonia and if this can help improve outcomes in critically ill patients with COVID-19.
   - We believe radiation will help improve the excess inflammation in the lungs that occurs as a complication of COVID-19.
   - The low dose radiation is much lower than is used in the treatment of other conditions and we believe it will not have significant side effects, and the risk will be much less than that of worsening Covid 19 pneumonia which can be life threatening.
   - 20 subjects will participate in this study.
2. **PROCEDURES**

Your participation will last approximately one month (28 days) measured from the date the radiation treatment is delivered. Procedures to determine if you are eligible to take part in a research study are called “screening” procedures. You must sign this informed consent form before any research procedures are done.

The procedures you will undergo during the screening period will include:

- A review of your medical history and confirmation of the Covid 19 infection.
- A review of any medication(s) you are currently taking.
- You will undergo pregnancy test if you are a woman of childbearing age.
- Blood tests to determine your blood, kidney and liver function
- Blood tests to determine some markers that indicate that your immune system is overactive
- Chest Imaging (x-ray or CT scan)

Most of these tests are being done as part of your routine care and will not need to be repeated. If the test results show that you are eligible to participate in the study, and you agree to receive this treatment, you will be enrolled on the study. If you do not meet the eligibility criteria, you will not be able to participate in the study.

If the screening procedures confirm that you are eligible to participate in the research study, you will be scheduled for the experimental treatment, which will consist of receiving a low dose of radiation to your entire lung, which is where the over active immune system is causing this life threatening inflammation. The effect of the low dose radiation is to destroy some of the immune cells that are contributing to this excessive inflammation. You will be transported to the radiotherapy department, where you will meet the staff involved with the radiation treatment. You will be asked to remove your shoes and all clothing from the waist up, and change into a gown. The radiation staff will tell you about all steps involved with the treatment. After explaining the process, you will receive low dose whole-lung radiation therapy once and then you will be monitored before being transported back to the ward.

If you are on a ventilator to support your breathing, you will continue to receive your breathing assistance through the ventilator during the transportation and during the radiation treatment.

Since you have a Covid 19 infection, all personnel accompanying you and helping administer the treatment will have full PPE to protect themselves against Covid 19 and also avoid spreading it to other individuals. The treatment delivery time is estimated to be only a few minutes while the entire time required for the treatment, including transportation and treatment set up may take up to 30 minutes.

The treatment provided to you is not a tablet or an infusion – it is invisible energy (X-Rays) similar to what you get during a chest xray or CT scan. However, this X-ray treatment is more powerful and delivered from a different radiation machine. It is delivered by a machine called a ‘linear accelerator’ in a specialized room called a “vault” that is surrounded by thick concrete walls to avoid any of the radiation escaping outside the vault

All evaluations and routine medical care and treatment provided by your regular hospital doctors and nurses will continue and your participation in this study will not interfere with all of that. The low dose radiation will be provided to you in addition to all of your routine care. If you or the team of doctors caring for you notice improvement or worsening of your status, they will intervene as they would regardless of your study participation. All patients participating in this trial will receive low dose radiation treatment

To determine the outcome (side effects or benefits) of the treatment, your study doctors together with the medical team will follow and monitor you closely over the next days after the treatment. The monitoring will include routine blood work, your vital signs, your oxygen blood level. This is in order to identify any side effects (or benefit) that you may experience during the first 24h and subsequently daily following your treatment.

Additional Blood samples, that we are referring to as “special Labs” will be used for analysis of how your immune system responds to the treatment.  These will be collected from you on days 1, 3,7, 14, 28 as part of this study. These samples will be stored for future testing and analysis and may need to be shipped outside Kenya if that technology is not available in Kenya. Your name or identification information will not be present on these stored samples; instead they will bear anonymized numbers or codes. We shall request permission from the hospital’s Research Ethics Committee (REC) i.e. Aga Khan University Hospital, Nairobi Institutional Ethical Research Committee (IERC) before we proceed with the analysis of these “special labs”.

1. **POSSIBLE RISKS OR DISCOMFORT**

There is little to no risk of you being exposed to any radiation above normal background radiation levels except for when the machine is intentionally turned on to deliver your treatment. Low-dose radiation therapy is a well-tolerated and well-studied treatment. It is unlikely to cause any acute toxicity other than fatigue, mild nausea and temporary drop in blood counts (which should have no impact on your health).

You or your next of kin, if applicable, will receive information on radiotherapy, including what it entails, the different types of radiotherapy and what to expect during radiotherapy, such as possible side effects and dietary specifications (if indicated). If you have any questions at any time, contact your study doctor (see section 11 below for contact information). Psychosocial counselling will also be available to you and your next of kin as part of our service to patients with covid 19 infection.

Radiation Safety: The main risk associated with radiation therapy is the possibility of developing a radiation induced cancer later in life. This risk mostly depends on age and dose of radiation. Children have the highest risk while the risk is lower in adults and decreases substantially with increasing age. The dose of radiation used in this study (100cGy) will be much lower than the radiation used to treat cancers in the lung. The risk of developing a secondary caner because of this low dose radiation is less than 1 in 100 (less than 1 %).

There may be a risk of exposing an unborn child to radiation. Women and men must take precautions to avoid exposing an unborn child. If you are pregnant or planning to become pregnant within 6 months of receiving radiotherapy, you cannot participate in this study. You must tell your study doctor if you become pregnant within 6 months of receiving radiotherapy.

1. **POSSIBLE BENEFITS**

There may be no direct benefit to your participation in this study since this is an experimental trial to determine safety of this treatment and whether we can safely deliver it to severely ill patients with Covid 19 infection. However, the aim is to understand if this kind of treatment can be delivered safely and potentially could benefit patients in the future. Through your participation we will learn and that knowledge may benefit others in the future. However, there is theoretical reason why this kind of treatment may be of benefit for patients with severe Covid 19 infection and your participation will allow us to determine the value of this form of treatment for patients with severe Covid 19 infection. Your condition may or may not improve as a result of your participation on this study. This study is designed to learn if COVID19 patients receiving radiation therapy may benefit from it. The study results may be used to help others in the future.

1. **FINANCIAL CONSIDERATIONS**
   - All other laboratory tests that will be used to determine your eligibility for this study will be performed as part of your routine care and these will be billed to your insurance or you.
   - You will not be required to pay any of the financial costs related to the radiation therapy and related PPE.
   - You will not be billed for any extra tests that will be done in the event of any side effects that are directly related to this low dose radiation therapy to the lung as determined by the investigator and team of experts assigned to review the causes.
2. **AVAILABLE TREATMENT ALTERNATIVES**

You can decide whether you want to be in this study or not. Your choice about participation will not affect your access to the standard medical care for your condition, but you will not be able to receive the investigational intervention (low-dose radiation therapy). If you decide to participate on this study, you will continue to receive appropriate medical care for your Covid 19 infection. The study involves only the addition of one dose of LDRT. All other medical management will be at the determination of your treating doctor and following acceptable best practice guidelines.

1. **AVAILABLE MEDICAL TREATMENT FOR ADVERSE EXPERIENCES**

This study involves minimal risk. Medical treatment for any study related adverse effects directly related to radiation will be provided to you at no cost.

1. **CONFIDENTIALITY**

Your identity in this study will be treated as confidential. The results of the study, including laboratory or any other data, may be published for scientific purposes but will not give your name or include any identifiable references to you. However, any records or data obtained as a result of your participation in this study may be reviewed by the sponsor or their representatives, AKU IERC members and Pharmacy and Poisons Board (PPB).

Your privacy will be maintained by removing any identifiers. There will be limited access to the details; access will be available to the research team only. Once data collection is complete, all identifiers will be removed from the data to maintain privacy.

1. **TERMINATION OF RESEARCH STUDY**

You are free to choose whether or not to participate in this study. There will be no penalty or loss of benefits to which you are otherwise entitled if you choose not to participate. You will be provided with any significant new findings developed during the course of this study that may relate to or influence your willingness to continue participation. In the event you decide to discontinue your participation in the study,

- - There are no potential consequences
  - You may notify your medical doctor or your study doctor about your decision to terminate your participation. In which case details of your treatment will closed and no treatment related follow up will occur.

Your participation in the study may be terminated by the investigator without your consent under certain circumstances that include risk to you or other participants, or unexpected side effects related to low dose lung radiation. Termination of study will not affect you if you have already received your low dose radiation treatment.

1. **AVAILABLE SOURCES OF INFORMATION**

**Person to contact for any questions:**

If you have any questions you can contact the Principal Investigator **Prof. Mansoor Saleh,** Cell : **0780428334**

email [Mansoor.saleh@aku.edu](mailto:Mansoor.saleh@aku.edu)

If you have questions related to your child’s rights as a research participant or if you have questions, concerns, input, or complaints relating to conduct of this study and you would like to speak to someone independent of the study, you may contact: call Aga Khan University Institutional Ethics Review Committee (a group of people who review the research to protect your rights) at **+254 20 366 2148/ +254 20 366 1136** email; [AKUKenya.ResearchOffice@aku.edu](mailto:AKUKenya.ResearchOffice@aku.edu)

**Person to contact in case of research related injury:**

If you are injured, you can contact: Day/Night emergency number 020 366 2022.

1. **AUTHORIZATION**

- I confirm that I have read this consent form or it has been read to me.
- I have understood this consent form, and I volunteer to participate in this research study.
- All my questions have been adequately answered
- I voluntarily choose to participate, but I understand that my consent does not take away any legal rights in the case of negligence or other legal fault of anyone who is involved in this study.
- I understand that I will receive a copy of this form

| Patient name (print) |  |  |
| --- | --- | --- |
| Patient signature |  | Date |
|  | | |
| Next of Kin Name (If applicable) |  | Relationship |
| Next of Kin signature |  | Date |
| **I, the undersigned, have fully explained this informed consent to the patient named above and/or the patient’s legally authorized representative.** | | |
|  | | |
| Name of person conducting informed consent discussion (print) |  |  |
|  | | |
| Signature of person conducting informed consent discussion |  | Date |
|  | | |
| Witness name ^a^ (print) |  |  |
|  | | |
| Witness signature ^a^ |  | Date |

# ***Appendix (ii)*** *Informed Consent form(Swahili)*

***Kiambatanisho (ii)*** *fomu ya idhini (Toleo la Kiswahili)*

| **Habari ya mradi** | |
| --- | --- |
| Kichwa cha mradi: | A pilot phase Ib/II study of whole-lung low dose radiation therapy (LDRT) in the treatment of severe Covid 19 pneumonia patients on (or requiring) mechanical ventilatory support. |
| Mpelelezi mkuu | Prof. Mansoor Saleh |
| Shirika | Aga Khan University Hospital, Nairobi |
| Mdhamini | Aga Khan University Hospital, Nairobi |
| **1. UTANGULIZI**  Jina langu ni Mansoor Saleh katika idara ya Hematology-Oncology katika Hospitali ya Chuo Kikuu cha Aga Khan Nairobi. Pamoja na wenzangu, tunafanya majaribio ya utafiti wa kliniki juu ya utumiaji wa tiba ya kipimo cha chini cha matibabu ya mionzi kwa matibabu kwa wagonjwa sana wenye ugonjwa wa COVID-19. Tutakuwa tunatibu vikundi 2 tofauti vya wagonjwa. Kikundi 1 watakuwa wagonjwa ambao kiwango cha oksijeni haiboreshi na mgonjwa anaweza kuhitaji kuwekwa kwenye kiingilizi (mashine ambayo hutoa oksijeni kwenye mapafu ya mgonjwa na inawasaidia kupumua) ikiwa mambo yatazidi. Kikundi 2 watakuwa wale wagonjwa ambao wako tayari kwenye kiingilizi kwa si zaidi ya siku 5.  COVID-19 ni janga la ulimwenguni pote linalosababishwa na virusi vya Korona. Husababisha homa, kikohozi na uchovu. Wakati wagonjwa wengi wanaonekana kupona, wagonjwa wengine hupata shida kali mara nyingi zinazohusisha mapafu na hawawezi kupumua na wanahitaji kupokea kiwango cha juu cha oksijeni au kuwekwa kwa kiingilizi ili kuwawezesha kuishi. Wakati kuna matibabu kadhaa ambayo yanajaribu kwa wagonjwa walio na pneumonia ya Covid, kuna wagonjwa wengi ambao wamemaliza chaguzi hizi au hawawezi kuzipokea. Hata chini ya hali nzuri, idadi ndogo ya wagonjwa huugua sana na hawawezi kupata oksijeni ya kutosha kuendeleza mwili wao. Chini ya hali hizo, wagonjwa wanaweza kuhitaji kupokea uingizaji hewa wa mitambo kupitia mashine ambayo itatoa oksijeni na kusaidia kupumua kwa mgonjwa mwenyewe.  Tiba inayotolewa ni kipimo moja cha mionzi kwa mapafu, ambapo virusi husababisha uharibifu mkubwa na kwa sababu wagonjwa wanashindwa kupumua na kupata oksijeni ya kutosha ndani ya miili yao. Inatokea kwamba mfumo wa kinga ya mgonjwa, ambao unapaswa kupigana na virusi umezidi na hutengeneza vitu vinavyosababisha mafuriko ya tishu za mapafu ambamo seli za kinga na maji huzuia uhuru wa mtiririko wa oksijeni kutoka kwa mapafu kuingia kwenye mfumo wa damu. Mfumo huu wa kinga kupita kiasi na mafuriko ya tishu za mapafu unaitwa dhoruba ya cytokine (cytokines ni vitu hivyo vinavyozalishwa na mfumo wa kinga wakati wa mapambano dhidi ya virusi). Dhoruba hii ya cytokine husababisha kile tunachokiita ARDS (ugonjwa wa kupumua kwa papo hapo), ambayo ni mafuriko ya tishu za mapafu na seli za kinga na maji ambayo husababisha tishu za mapafu kuwa na shida na kutoweza kuhamisha oksijeni kutoka kwa hewa kuingia kwenye mfumo wa damu. Kwenye X-ray au skani ya CT, ARDS huonekana kama "nzito wa wamingu kwa mapafu".  Kabla ya matumizi ya dawa za kutibu nimonia, mionzi mara nyingi ilitumiwa kama moja wapo ya matibabu ya kusaidia wagonjwa walio na virusi vya nimonia. Dozi ya chini ya mionzi pia imetumika kutibu magonjwa kadhaa ambayo hutokana na shughuli nyingi za mfumo wa kinga. Inawezekana kwamba mkakati huu huo wa mionzi ya kipimo cha chini unaweza kutumika kutibu nimonia, dhoruba ya cytokine na ARDS (kuvimba kali kwa mapafu) iliyosababishwa na COVID-19. Mionzi ya dozi ya chini kwa mapafu inastahili kuwa nzuri katika kupunguza uchochezi unaosababishwa na virusi na inaweza kusaidia kuboresha matokeo kwa walio wagonjwa sana.   1. **MALENGO YA UTAFITI HUU**   o Unaulizwa kushiriki katika utafiti ulioundwa ili kujua ikiwa tiba ya kiwango cha chini cha mionzi inaweza kudhibitiwa kwa usalama kwa mgonjwa aliye na nimonia kali ya Covid 19 na ikiwa hii inaweza kusaidia kuboresha matokeo kwa walio wagonjwa sana na COVID-19.  o Tunaamini kuwa mionzi itasaidia kuboresha uchochezi mwingi katika mapafu ambayo hufanyika kama shida ya COVID-19.  o Mionzi ya kipimo cha chini ni ya chini sana kuliko inayotumika katika matibabu ya hali zingine na tunaamini haitakuwa na athari kubwa, na hatari itakuwa chini ya ile ya ugonjwa wa nimonia mbaya ya Covid 19 ambayo inaweza kutishia maisha.  o Washiriki 20 watashiriki katika utafiti huu.  Mamlaka zinazoidhinisha utafiti huu ni:  • Kamati ya Kupitia Maadili ya Taasisi ya Chuo Kikuu cha Aga Khan (IERC)  • Bodi ya Dawa na Sumu (PPB)  • Baraza la Kitaifa la Sayansi, Teknolojia na Ubunifu (NACOSTI)   1. **TARATIBU**   Ushiriki wako utaendelea takriban mwezi mmoja (siku 28) uliopimwa kutoka tarehe matibabu ya mionzi itakapopeanwa. Taratibu za kuamua ikiwa unastahili kushiriki katika utafiti unaitwa taratibu za "uchunguzi". Lazima utatia sahihi kwa fomu hii ya idhini kabla ya mchakato wowote wa utafiti kufanywa  Taratibu utapitia wakati wa uchunguzi ni pamoja na:  • Mapitio ya historia yako ya matibabu na uthibitisho wa maambukizo ya Covid 19.  • Hakiki ya dawa yoyote unayotumia sasa.  • Utafanya upimaji wa ujauzito ikiwa wewe ni mwanamke wa umri wa kuzaa.   - Uchunguzi wa damu ili kuamua damu yako, utendakazi wa figo na ini - Uchunguzi wa damu ili kuona alama kadhaa ambazo zinaonyesha kuwa mfumo wako wa kinga ni mzito - Kuiga Kifua (x-ray au skani ya CT)  1. **TARATIBU**   Vipimo vingi inafanywa kama sehemu ya utunzaji wako wa kawaida na haitahitaji kurudiwa. Ikiwa matokeo ya majaribio yanaonyesha kuwa unastahili kushiriki katika utafiti, na unakubali kupokea matibabu haya, utaandikishwa kwenye utafiti. Ikiwa hautafikia vigezo vya kustahiki, hautaweza kushiriki kwenye utafiti.  Ikiwa michakato ya uchunguzi inathibitisha kuwa unastahili kushiriki katika utafiti, utapangwa kwa matibabu ya majaribio, ambayo yatakuwa na kupokea kipimo kidogo cha mionzi kwa mapafu yako yote, ambapo nguvu ya kinga ya mwili inasababisha uvimbe wa kutishia maisha. Athari za mionzi ya kipimo cha chini ni kuharibu baadhi ya seli za kinga ambazo zinachangia uchochezi huu mwingi. Dozi ya chini ya mionzi kwa mapafu yote utapewa mara moja na utasafirishwa kwa kitengo cha tiba ya mionzi na kisha kurudishwa kwenye kitengo chako.  Utasafirishwa kwenda kwa idara ya tiba ya mionzi, ambapo utakutana na wafanyikazi wanaohusika na matibabu ya mionzi. Utaulizwa uvue viatu vyako na nguo zote kutoka kiunoni kwenda juu, na ubadilishe kanzu. Wafanyikazi wa mionzi watakuambia juu ya hatua zote zinazohusika na matibabu. Baada ya kuelezea mchakato huo, Utapokea kipimo cha chini tiba ya mionzi ya mapafu utapewa mara moja na kisha utafuatiliwa kabla ya kusafirishwa kurudi kwenye kitengo cha tiba ya mionzi na kisha kurudi kwenye kitengo chako.  Ikiwa uko kwenye kiingilizi cha kusaidia kupumua kwako, utaendelea kupokea msaada wako wa kupumua kupitia kiingilizi wakati wa usafirishaji na wakati wa matibabu ya mionzi. Kwa kuwa una maambukizo ya Covid 19, wafanyikazi wote wanaoongozana na wewe na kusaidia kudhibiti matibabu watakuwa na PPE kamili ya kujikinga dhidi ya Covid 19 na pia kwa kuepuka kuieneza kwa watu wengine. Wakati wa utoaji wa matibabu inakadiriwa kuwa dakika chache wakati wakati wote unahitajika kwa matibabu, pamoja na usafirishaji na matibabu yaliyowekwa yanaweza kuchukua hadi dakika 30.  Matibabu uliyopewa sio kidonge au infusion - ni nishati isiyoonekana (X-Rays) sawa na ile unayopata wakati wa X-ray ya kifua au skani ya CT. Walakini, matibabu haya ya X-ray ni ya nguvu zaidi na hutolewa kutoka mashine tofauti ya mionzi. Inawasilishwa na mashine inayoitwa 'linear accelerator' katika chumba maalumu kinachoitwa "vault" ambayo imezungukwa na ukuta mnene wa zege ili kuepusha mionzi yoyote inayoweza kutoroka nje ya ukumbi  Tathmini zote na matibabu ya kawaida na matibabu yanayotolewa na madaktari na wauguzi wa hospitali yako ya kawaida yataendelea na ushiriki wako katika utafiti huu hautazuia chochote. Mionzi ya kipimo cha chini itatolewa kwako kwa kuongezea utunzaji wako wa kawaida. Ikiwa wewe au timu ya madaktari wanaokujali hugundua uboreshaji au kudhoofika kwa hali yako, wataingilia kati kama wangefanya bila kujali ushiriki wako kwa utafiti. Wagonjwa wote wanaoshiriki katika jaribio hili watapata matibabu ya kiwango cha chini cha mionzi  Kuamua matokeo (athari au faida) za matibabu, madaktari wako wa utafiti pamoja na timu ya matibabu watafuata na kukufuatilia kwa karibu siku zijazo baada ya matibabu. Ufuatiliaji utajumuisha kazi ya kawaida ya damu, ishara zako muhimu, kiwango chako cha damu ya oksijeni. Hii ni ili kubaini athari yoyote (au faida) ambayo unaweza kupata wakati wa masaa 24 ya kwanza na baadaye kila siku kufuatia matibabu yako.  Sampuli za ziada za Damu, ambazo tunazitaja kama "Maabara maalum" zitatumika kwa uchambuzi wa jinsi mfumo wako wa kinga unavyojibu matibabu. Hizi zitakusanywa kutoka kwako siku ya 1, 3,7, 14, 28 kama sehemu ya utafiti huu. Sampuli hizi zitahifadhiwa kwa upimaji na uchambuzi kwa siku zijazo na zinaweza kuhitaji kusafirishwa nje ya Kenya ikiwa teknolojia hiyo haipatikani Kenya. Jina lako au habari ya kukutambulisha haitakuwapo kwenye sampuli hizi zilizohifadhiwa; badala yake watabeba nambari au nambari zisizojulikana. Tutaomba ruhusa kutoka kwa Kamati ya Maadili ya Utafiti ya hospitali (REC) yaani Hospitali ya Chuo Kikuu cha Aga Khan, Kamati ya Utafiti wa Maadili ya Taasisi ya Nairobi (IERC) kabla ya kuendelea na uchambuzi wa hizi "maabara maalum"  4**. HATARI ZINAOWEZEKANA AU USUMBUFU**  Kuna hatari kidogo kwa kusipokuwa nayo kwako wewe kuwekwa kwenye mionzi zaidi ya viwango vya kawaida isipokuwa wakati mashine imewashwa kwa makusudi ya kutoa matibabu yako. Tiba ya mionzi ya kiwango cha chini ni matibabu ya kuvumiliwa vizuri na matibabu yametafitiwa vizuri. Haiwezekani kusababisha sumu yoyote kali mabali na uchovu, kichefuchefu kali na kushuka kwa muda kwa hesabu za damu (ambayo haifai kuwa na athari kwa afya yako).  Wewe au ukoo wako wa karibu, ikiwa inafaa, atapokea habari juu ya radiotherapy, pamoja na kile kinachojumuisha, aina tofauti za radiotherapy na nini cha kutarajia wakati wa radiotherapy, kama vile athari zinazowezekana na maelezo ya lishe (ikiwa imeonyeshwa). Ikiwa una maswali yoyote wakati wowote, wasiliana na daktari wako wa utafiti (angalia sehemu ya 11 hapa chini kwa habari ya mawasiliano). Ushauri wa kisaikolojia pia utapatikana kwako na jamaa yako kama sehemu ya huduma yetu kwa wagonjwa walio na maambukizo ya covid 19.  Usalama wa mionzi: Hatari kuu inayohusishwa na tiba ya mionzi ni uwezekano wa kupata saratani iliyosababishwa na mionzi baadaye katika maisha. Hatari hii inategemea umri na kipimo cha mionzi. Watoto wana hatari kubwa wakati hatari iko chini kwa watu wazima na hupungua sana na miaka inapoongezeka. Kiwango cha mionzi inayotumika katika utafiti huu (100cGy) itakuwa chini sana kuliko mionzi inayotumika kutibu saratani kwenye mapafu. Hatari ya kukuza saratani ya sekondari kwa sababu ya mionzi ya kipimo cha chini ni chini ya 1 kwa 100 (chini ya 1%).  Kunaweza kuwa na hatari ya kufunua mtoto ambaye hajazaliwa kwa mionzi. Wanawake na wanaume lazima wachukue tahadhari ili kuepuka kufunua mtoto ambaye hajazaliwa. Ikiwa una mjamzito au unapanga kuwa mjamzito ndani ya miezi 6 ya kupokea radiotherapy, huwezi kushiriki katika utafiti huu. Lazima umwambie daktari wako wa masomo ikiwa utapata mjamzito ndani ya miezi 6 ya kupokea radiotherapy.  **5. FAIDA INAYOWEZEKANA**  Hakuwezi kuwa na faida ya moja kwa moja kwa kushiriki kwako katika utafiti huu kwani hii ni jaribio la majaribio kuamua usalama wa matibabu hii na ikiwa tunaweza kuipeana kwa wagonjwa wenye ugonjwa wa Covid 19 kwa usalama. Walakini, lengo ni kuelewa ikiwa matibabu ya aina hii yanaweza kutolewa kwa usalama na uwezekano wa kufaidisha kwa wagonjwa katika siku zijazo. Kupitia ushiriki wako tutajifunza na kwamba maarifa yanaweza kuwanufaisha wengine katika siku zijazo. Walakini, kuna sababu ya nadharia kwa nini aina hii ya matibabu inaweza kuwa ya faida kwa wagonjwa walio na maambukizi kali ya Covid 19 na kushiriki kwako kutaturuhusu kutamua thamani ya njia hii ya matibabu kwa wagonjwa walio na maambukizi kali ya Covid 19. Hali yako inaweza au haiwezi kuboreka kutokana na ushiriki wako kwenye utafiti huu. Utafiti huu umeundwa kujifunza ikiwa wagonjwa wa COVID19 wanaopokea tiba ya mionzi wanaweza kufaidika nayo. Matokeo ya utafiti yanaweza kutumiwa kusaidia wengine katika siku zijazo.  **6. KUZINGATIA FEDHA**  o Hautalazimika kulipa gharama zozote za kifedha zinazohusiana na tiba ya mionzi na PPE inayohusiana.  o Vipimo vingine vyote vya maabara ambavyo vitatumika kuamua kustahiki kwako kwa utafiti huu vitafanywa kama sehemu ya utunzaji wako wa kawaida na hizi zitatozwa kwa bima yako au wewe.  o Hutatozwa malipo yoyote ya ziada ambayo yatafanyika katika tukio la athari zozote ambazo zinahusiana moja kwa moja na tiba hii ya kiwango cha chini cha mionzi kwa mapafu.  **7. KUPATIKANA KWA MATIBABU MBADALA**  Unaweza kuamua ikiwa unataka kuwa katika utafiti huu au la. Chaguo lako juu la kushiriki haitaathiri ufikiaji wako katika huduma ya matibabu ya hali yako, lakini hautaweza kupokea uchunguzi wa uingiliaji (tiba ya mionzi ya kiwango cha chini). Ukiamua kushiriki kwenye utafiti huu, utaendelea kupata huduma sahihi ya matibabu kwa maambukizi yako ya Covid 19. Utafiti huu unajumuisha kuongeza tu kwa kipimo kikuu cha LDRT. Usimamizi mwingine wote wa matibabu utakuwa kwa uamuzi wa daktari wako wa kutibu na kutafuata miongozo bora inayokubalika.  **8. KUPATIKANA KWA TIBA YA MATIBABU KWA MATUKIO MABAYA**  Utafiti huu unajumuisha hatari ndogo. Tiba ya matibabu kwa athari mbaya yoyote inayohusiana na utafiti inayohusiana na mionzi itapewa bure.  **9. USIRI**  Kitambulisho chako katika utafiti huu itawekwa kuwa siri. Matokeo ya utafiti huo, pamoja na maabara au data nyingine yoyote, inaweza kuchapishwa kwa sababu za kisayansi lakini haitatoa jina lako au kujumuisha marejeleo yoyote yanayotambulika kwako. Walakini, rekodi yoyote au data iliyopatikana kwa sababu ya kushiriki kwako katika utafiti huu inaweza kupitiwa na mdhamini au wawakilishi wao, au na wanachama wa AKU IERC na Bodi ya Dawa na Sumu (PPB).  Usiri wako utatunzwa kwa kuondoa vitambulisho vyovyote. Kutakuwa na ufikiaji mdogo wa maelezo; ufikiaji utapatikana kwa timu ya utafiti tu. Mara mkusanyiko wa data utakapokamilika, vitambulisho vyote vitaondolewa kutoka kwa data ili kuweka faragha.  **10. KUMALIZIKA KWA UTAFITI**  Uko huru kuchagua ikiwa au sivyo kwa kushiriki katika utafiti huu. Hakutakuwa na adhabu au upotezaji wa faida ambazo kwa haki vingenevyo ikiwa utachagua kutoshiriki. Utapewa matokeo yoyote muhimu yanayopatikana wakati wa utafiti huu ambayo yanaweza kuhusiana na au kushawishi nia yako ya kuendelea kushiriki. Katika tukio ambalo utaamua kuacha kushiriki kwako kwenye utafiti,  o Hakuna uwezo wa majuto yeyote  o Unaweza kumweleza daktari wako wa matibabu au daktari wako wa utafiti juu ya uamuzi wako wa kumaliza ushiriki wako. Katika hali hii maelezo ya matibabu yako yatafungwa na hakuna matibabu yoyote yanayofuata yatatokea.  Ushiriki wako katika utafiti unaweza kusitishwa na mpelelezi bila idhini yako chini ya hali fulani ambazo zinajumuisha hatari kwako au washiriki wengine, au athari mbaya zisizotarajiwa zinazohusiana kiwango cha chini cha mionzi ya mapafu. Kukomesha utafiti hakutakuathiri ikiwa tayari umepokea matibabu ya mionzi ya kiwango cha chini.  **11. KUPATIKANA KWA VYANZO VYA HABARI**  Mtu wa kuwasiliana kwa maswali yoyote:  Ikiwa una maswali yoyote unaweza kuwasiliana na Mpelelezi Mkuu **Prof Mansoor Saleh**, Simu: 0780428334  barua pepe [Mansoor.saleh@aku.edu](mailto:Mansoor.saleh@aku.edu)  Ikiwa una maswali yanayohusiana na haki za mtoto wako kama mshiriki wa utafiti au ikiwa una maswali, wasiwasi, pembejeo, au malalamiko yanayohusiana na mwenendo wa utafiti huu na ungependa kuongea na mtu huru wa masomo, unaweza kuwasiliana na: Kamati ya Maadili ya Taasisi ya Maadili ya Chuo Kikuu cha Aga Khan (kikundi cha watu ambao wanakagua utafiti ili kulinda haki zako) kwa **+254 20 366 2148 / +254 20 366 1136**; barua pepe; [AKUKenya.ResearchOffice@aku.edu](mailto:AKUKenya.ResearchOffice@aku.edu)  **Mtu wa kuwasiliana naye katika ikiwa kutakuwa na jeraha linalohusiana na utafiti:**  Ikiwa umeumia, unaweza kuwasiliana na: Nambari ya dharura ya Siku / Usiku 020 366 2022.  12. **IDHINI**  • Ninathibitisha kwamba nimesoma fomu hii ya idhini au nimesomewa.  • Ninaelewa fomu hii ya idhini, na nimejitolea kushiriki katika utafiti huu.  • Maswali yangu yote yamejibiwa vya kutosha  • Nachagua kushiriki kwa hiari, lakini ninaelewa kuwa idhini yangu haitoi haki yoyote ya kisheria katika iwapo uzembe au kosa lolote lingine la kisheria kwa mtu yeyote anayehusika katika utafiti huu.  • Ninaelewa kuwa nitapokea nakala ya fomu hii  _________________________________________________________  Jina la mgonjwa (chapisha)  ___________________________________________ __________________________  Sahihi ya mgonjwa Tarehe  ________________________________________ ___________________________  Jina la jamaa (Ikiwa inatumika) Uhusiano  ________________________________________ _________________________  Sahihi ya jamaa Tarehe  **Mimi, mwenye kuweka sahihi, nimeelezewa kikamilifu idhini hii ya habari kwa mgonjwa aliyetajwa hapo juu na / au mwakilishi aliyeidhinishwa kisheria.**  _____________________________________________ __________________________  Jina la mtu anayeendesha mchakato wa idhini (chapisha) Tarehe  _____________________________________________  Sahihi ya mtu anayeendesha makubaliano ya idhini    Jina la Shahidi (kuchapisha)___________________________________________  Sahihi ya Shahidi ^______________________________________________^ Tarehe_____________________ | |

# ***Appendix (iii)*** *AKUH, N Inpatient COVID 19 management protocol*

# ***Appendix (iv)*** *Case Report Form*

|  | **PATIENT DEMOGRAPHICS** | | | | | | | | | | | | | | | | | | | | | | | |  | | |  | | |  | | |  | | |  | | |  | | |  | | | | |  | | | | |  | |  | |  | |  | |  | |  | |  | |  |  |  |  |  |  |  |
| --- | --- | --- | --- | --- | --- | --- | --- | --- | --- | --- | --- | --- | --- | --- | --- | --- | --- | --- | --- | --- | --- | --- | --- | --- | --- | --- | --- | --- | --- | --- | --- | --- | --- | --- | --- | --- | --- | --- | --- | --- | --- | --- | --- | --- | --- | --- | --- | --- | --- | --- | --- | --- | --- | --- | --- | --- | --- | --- | --- | --- | --- | --- | --- | --- | --- | --- | --- | --- | --- | --- | --- | --- | --- |
|  |  | |  | |  |  |  |  |  |  |  |  | |  | | | | | | | | | | | D | | | D | | | M | | | M | | | Y | | | Y | | | Y | | | | | Y | | | | |  | |  | |  | |  | |  | |  | |  | |  |  |  |  |  |  |  |
|  | **Day month and year of birth** | | | | | | | | | |  |  | |  | | | | | | | | | | |  | | |  | | |  | | |  | | |  | | |  | | |  | | | | |  | | | | |  | |  | |  | |  | |  | |  | |  | |  |  |  |  |  |  |  |
|  |  | |  | |  |  |  |  |  |  |  |  | |  | | | | | | | | | | |  | | |  | | |  | | |  | | |  | | |  | | |  | | | | |  | | | | |  | |  | |  | |  | |  | |  | |  | |  |  |  |  |  |  |  |
|  | **Age** | |  | |  |  | YEARS | |  |  |  |  | |  | | | | | | | | | | |  | | |  | | |  | | |  | | |  | | |  | | |  | | | | |  | | | | |  | |  | |  | |  | |  | |  | |  | |  |  |  |  |  |  |  |
|  |  | |  | |  |  |  |  |  |  |  |  | |  | | | | | | | | | | |  | | |  | | |  | | |  | | |  | | |  | | |  | | | | |  | | | | |  | |  | |  | |  | |  | |  | |  | |  |  |  |  |  |  |  |
|  | **Sex** | |  | | Male …………. | | | 1 |  |  |  |  | |  | | | | | | | | | | |  | | |  | | |  | | |  | | |  | | |  | | |  | | | | |  | | | | |  | |  | |  | |  | |  | |  | |  | |  |  |  |  |  |  |  |
|  |  | |  | | Female…………………… | | | 2 |  |  |  |  | |  | | | | | | | | | | |  | | |  | | |  | | |  | | |  | | |  | | |  | | | | |  | | | | |  | |  | |  | |  | |  | |  | |  | |  |  |  |  |  |  |  |
|  |  | **Race** | | (Check Appropriate Option) | | | | | | | | |  | | |  | |  | |  | |  | |  | | |  | | |  | | |  | | |  | | |  | | |  | | |  | |  | | |  | |  | |  | |  | |  | |  | |  | |  | |  |  |  |  |  |  |  |  |
|  | Black African………………………………………………………………. | | | | | | | | | | | | | | 1 | |  | |  | |  | |  | | |  | | |  | | |  | | |  | | |  | | |  | | |  | |  | | |  | |  | | |  | |  | |  | |  | | | | | | | | | | | | | |
|  | Asian……………………………………………………………………………………………….. | | | | | | | | | | | | | | 2 | |  | |  | |  | |  | | |  | | |  | | |  | | |  | | |  | | |  | | |  | |  | | |  | |  | | |  | |  | |  | |  |  |  |  |  |  |  |  |  |  |  |  |  |  |
|  | Caucasian……………………………………………………………………………………………. | | | | | | | | | | | | | | 3 | |  | |  | |  | |  | | |  | | |  | | |  | | |  | | |  | | |  | | |  | |  | | |  | |  | | |  | |  | |  | |  |  |  |  |  |  |  |  |  |  |  |  |  |  |
|  | Unknown……………………………………………………………. | | | | | | | | | | | | | | 4 | |  | |  | |  | |  | | |  | | |  | | |  | | |  | | |  | | |  | | |  | |  | | |  | |  | | |  | |  | |  | |  |  |  |  |  |  |  |  |  |  |  |  |  |  |

|  | **Smoking history** | | | |  | Never |  | 1 |  |  |  |  |  |  |  |  |  |  |  |  |  |  |  |  |  |  |  |  |  |  |  |  |
| --- | --- | --- | --- | --- | --- | --- | --- | --- | --- | --- | --- | --- | --- | --- | --- | --- | --- | --- | --- | --- | --- | --- | --- | --- | --- | --- | --- | --- | --- | --- | --- | --- |
|  |  |  |  |  |  | Current | | 2 |  |  |  |  |  |  |  |  |  |  |  |  |  |  |  |  |  |  |  |  |  |  |  |  |
|  |  |  |  |  |  | Former | | 3 |  |  |  |  |  |  |  |  |  |  |  |  |  |  |  |  |  |  |  |  |  |  |  |  |

| **If current or former user, specify below.** | | | | | | | | Otherwise, N/A | | |  |  | |  |  |  |  |  |  |  |  |  |  |  |  |  |  |  |  |  |  |  |  |  |  |  |
| --- | --- | --- | --- | --- | --- | --- | --- | --- | --- | --- | --- | --- | --- | --- | --- | --- | --- | --- | --- | --- | --- | --- | --- | --- | --- | --- | --- | --- | --- | --- | --- | --- | --- | --- | --- | --- |
|  | Month and year subject last smoked | | | | | | | | | | | | M | M | M | Y | Y | Y | Y |  |  |  |  | | | |  |  |  |  |  |  |  |  |  |  |
|  |  | | | | | | | | | | | | |  |  |  |  |  | |  |  |  |  |  |  |  |  |  |  |  |  |  |  |  |  |  |
|  | Total number of years subject smoked | | | | | | | | | | | | |  |  |  |  | Years | |  |  |  |  |  |  |  |  |  |  |  |  |  |  |  |  |  |
|  |  |  |  |  |  |  |  |  |  |  | | |  |  |  |  |  |  |  |  |  |  |  |  |  |  |  |  |  |  |  |  |  |  |  |  |
|  |  |  |  |  |  |  |  |  |  | YES…. | | |  |  |  |  | | |  | 1 |  |  |  |  |  |  |  |  |  |  |  |  |  |  |  |  |
|  | Does subject use e-cigarettes? | | | | | | | | | NO | | |  |  |  |  | | |  | 2 |  |  |  |  |  |  |  |  |  |  |  |  |  |  |  |  |
|  |  |  |  |  |  |  |  |  |  |  | | |  |  |  |  |  |  |  |  |  |  |  |  |  |  |  |  |  |  |  |  |  |  |  |  |
|  |  |  |  |  |  |  |  |  |  |  | | |  |  |  |  |  |  |  |  |  |  |  |  |  |  |  |  |  |  |  |  |  |  |  |  |

| Physical Examination at Baseline | | | | | | | | | | |
| --- | --- | --- | --- | --- | --- | --- | --- | --- | --- | --- |
| (Please tick) | | | | | | | | | | |
| Code | System | Normal | Abnormal | N/A |  | Code | System | Normal | Abnormal | N/A |
| 1 | Cardiovascular |  |  |  |  | 9 | Neoplasia |  |  |  |
| 2 | Respiratory |  |  |  |  | 10 | Neurological |  |  |  |
| 3 | Hepato-biliary |  |  |  |  | 11 | Psychological |  |  |  |
| 4 | Gastro-intestinal |  |  |  |  | 12 | Immunological |  |  |  |
| 5 | Genito-urinary |  |  |  |  | 13 | Dermatological |  |  |  |
| 6 | Endocrine |  |  |  |  | 14 | Allergies |  |  |  |
| 7 | Haematological |  |  |  |  | 15 | Eyes, ear, nose, throat |  |  |  |
| 8 | Musculo-skeletal |  |  |  |  | 00 | Other_____________ |  |  |  |

If ***abnormal*** for any of the above, give further details on the Baseline Conditions/AE Log. Use a separate line for each condition.

Comments: ____________________________________________________________________

______________________________________________________________________________

______________________________________________________________________________

Investigator: **______________ _____________________ ___________________**­­

Name Signature Date

| D | D | M | M | M | Y | Y | Y | Y |
| --- | --- | --- | --- | --- | --- | --- | --- | --- |

Date of first COVID-19 symptoms

Symptoms at time of diagnosis

| **Symptoms at time of diagnosis** | **YES** | **NO** |
| --- | --- | --- |
| Fever |  |  |
| Chills |  |  |
| Repeated shaking with chills |  |  |
| Cough |  |  |
| Persistent pain or pressure in chest |  |  |
| Shortness of breath or trouble breathing |  |  |
| GI symptoms (e.g., diarrhea, nausea, loss of appetite) |  |  |
| Loss of taste or smell |  |  |
| Sore throat |  |  |
| Headache |  |  |
| Fatigue |  |  |
| Confusion or inability to arouse |  |  |
| Muscle Pain |  |  |
| Bluish lips or face |  |  |
| Other, if other specify: ______________________ |  |  |
| Other, if other specify: ______________________ |  |  |

| **Targeted Medical History and Baseline conditions log**  **(Please tick)** | | | | | |
| --- | --- | --- | --- | --- | --- |
|  | **Medical Condition** | **Absent** | **Present** | **Start Date**  **(if present)** | **Stop Date**  **(if present) or**  **✔ if ongoing and add to Baseline/AE Log** |
| 1 | Diabetes |  |  |  |  |
| 2 | Hypertension |  |  |  |  |
| 3 | Hyperlipidemia |  |  |  |  |
| 4 | Asthma |  |  |  |  |
| 5 | COPD |  |  |  |  |
| 6 | Obesity |  |  |  |  |
| 7 | Myocardial Infarction |  |  |  |  |
| 8 | Atrial fibrillation |  |  |  |  |
| 9 | Stroke |  |  |  |  |
| 10 | Diverticulitis |  |  |  |  |
| 11 | GI Perforation |  |  |  |  |

| General Medical History and Baseline Conditions *other than those above* | | | | | | | | |
| --- | --- | --- | --- | --- | --- | --- | --- | --- |
| **Does the subject have any other medical history or baseline conditions? YES**    **NO** | | | | | | | | |
| Code | System | *Yes | No |  | Code | System | *Yes | No |
| 1 | Cardiovascular |  |  |  | 9 | Neoplasia |  |  |
| 2 | Respiratory |  |  |  | 10 | Neurological |  |  |
| 3 | Hepato-biliary |  |  |  | 11 | Psychological |  |  |
| 4 | Gastro-intestinal |  |  |  | 12 | Immunological |  |  |
| 5 | Genito-urinary |  |  |  | 13 | Dermatological |  |  |
| 6 | Endocrine |  |  |  | 14 | Allergies |  |  |
| 7 | Haematological |  |  |  | 15 | Eyes, ear, nose, throat |  |  |
| 8 | Musculo-skeletal |  |  |  | 00 | Other __________________ |  |  |

| **Surgery and Procedure History** |
| --- |
| Has the subject had ant relevant surgeries or procedure? YES  NO  If Yes,   \| **Surgery of procedure description** \| **Surgery or procedure date** \| \| --- \| --- \| \|  \|  \| \|  \|  \| \|  \|  \| \|  \|  \| \|  \|  \| \|  \|  \| \|  \|  \| \|  \|  \| \|  \|  \| \|  \|  \| \|  \|  \| |

**Baseline Conditions & Adverse Event Tracking Log *Check box if there were no adverse events to be recorded* 🞏**

| Date Reported | Adverse Event Description | Serious  (Y or N) | Start Date  **& Time** | End Date **& Time**  or ✔ if ongoing | Grade (CTCAE v.5) | Severity^2^ | Outcome^1^ | AE Treatment^3^ | Study Attribution^5^ | Action Taken**^4^** | Drug Attribution^5^^ | PI Initials | Date of PI Initials |
| --- | --- | --- | --- | --- | --- | --- | --- | --- | --- | --- | --- | --- | --- |
|  |  |  |  |  |  |  |  |  |  |  |  |  |  |
|  |  |  |  |  |  |  |  |  |  |  |  |  |  |
|  |  |  |  |  |  |  |  |  |  |  |  |  |  |
|  |  |  |  |  |  |  |  |  |  |  |  |  |  |

# - AE number. “1” indicates the first adverse event documented on the form, 2 = the second, etc. If the adverse event changes in severity, enter it as a separate adverse event row on the paper form using the same AE number as the one that ended.

| **Outcome^1^** | **Severity^2^** | **AE Treatment^3^** | **Action Taken^4^ with Study Intervention** | **Attribution/  Relatedness^5^** |
| --- | --- | --- | --- | --- |
|  | 1 – Mild |  | 1 – Interrupted | 1 – Definite |
| 1 – Not recovered/not resolved | 2 – Moderate | 1 – Medication(s) | 2 – Discontinued | 2 – Probable |
| 2 – Recovered w/sequelae | 3 – Severe | 2 – Non-medication TX | 3 – Dose reduced | 3 – Possible |
| 3 – Recovered w/o sequelae |  | 3 – None | 4 – Dose increased | 4 – Unlikely |
| 4 – Recovering/Resolving |  |  | 5 – Not Applicable | 5 – Unrelated |
| 5 – Fatal |  |  | 0 – None | 6 – Not Applicable (did not receive intervention) |

**Fertility Status Form**

**Female Fertility Status**

| Age: _________ | Reseponse |
| --- | --- |
| 1. Is the patient breastfeeding? | NO  YES |
| 1. child bearing potential? |  |
| 1. Post menopaUsal?   If yes, indicate date of Last Menstrual period (must be at least 12 months prior) and add to medical history and skip remaining questions. | NO  YES, Date of last menstrual  period: _____________ |
| 1. surgical procedures? if yes, indicate date and type (removal of ovaries, fallopian tubes, and/or uterus). add to medical history and skip remaining questions. | NO  YES, Date of procedure: _____________ |
| 1. Are you on any birth control? (Yes or No) If yes, indicate TYPE (oral hormonal contraceptive, injectable, implant, barrier, etc.) and start date.   add to concomittant medications. | NO  YES |
| 1. patient agrees to remain on birth control throughout the study and for 3 months after the last dose. |  |
| or  patient agrees to abstain from heterosexual sex throughout the study and for 3 months after the last dose. |  |

**ORDINAL SCALE RECORDING**

|  | Select the patient status based on the ordinal scale. The ordinal scale categories are as follows: | **Select one (✔)** |
| --- | --- | --- |
| 1 | Discharged (or “ready for discharge” as evidenced by normal body temperature and respiratory rate, and stable oxygen saturation on ambient air or ≤2L supplemental oxygen) |  |
| 2 | Non-ICU hospital ward (or “ready for hospital ward”) not requiring supplemental oxygen |  |
| 3 | Non-ICU hospital ward (or “ready for hospital ward”) requiring supplemental oxygen |  |
| 4 | ICU or non-ICU hospital ward, requiring non-invasive ventilation or high-flow oxygen |  |
| 5 | ICU, requiring intubation and mechanical ventilation |  |
| 6 | ICU, requiring ECMO or mechanical ventilation and additional organ support (e.g. vasopressors, renal replacement therapy) |  |
| 7 | Death |  |

Note:

- Patients who are ready to be discharged (e.g., still hospitalized due to non-medical or administrative reasons) will be assigned an ordinal scale of 1
- Patients in non-ICU hospital ward that are eligible for ICU care based on clinical presentation but awaiting ICU care will be assigned an ordinal scale of 4.
- Patients in ICU for administrative or non-medical reasons, who are ready for a non-ICU hospital ward, will be assigned an ordinal scale of 2 (if not requiring supplemental oxygen/non-invasive ventilation), 3 (requiring supplemental oxygen/ non-invasive ventilation), or 4 (if requiring non-invasive ventilation or high-flow oxygen).

**Investigator ________________________ __________________ ___________________**­­

Printed Name Signature Date

**Completed by ________________________ __________________ ___________________**­­

Printed Name Signature Date

**TREATMENT MONITORING CHART**

**PRE-DOSE (Day 1)**

| **Time-point** | **Actual Time** | **BP** | **TEMP** | **Pulse** | **RR** | **SPO2** | **O2 (L)** | **Comments (AEs, etc.)** |
| --- | --- | --- | --- | --- | --- | --- | --- | --- |
|  |  | mmhg | C | b/min | b/min | % |  |  |

**POST-DOSE (Day 1)**

| **Time-point** | **Actual Time** | **BP** | **TEMP** | **Pulse** | **RR** | **SPO2** | **O2 (L)** | **Comments (AEs, etc.)** |
| --- | --- | --- | --- | --- | --- | --- | --- | --- |
|  |  | mmhg | C | b/min | b/min | % |  |  |

**Daily Monitoring Visits**

| DATE   \| D \| D \| M \| M \| M \| Y \| Y \| Y \| Y \| \| --- \| --- \| --- \| --- \| --- \| --- \| --- \| --- \| --- \|   Date | VISIT DAY ___________________ |
| --- | --- | --- | --- | --- | --- | --- | --- | --- | --- | --- |
| Time of assessment (24hrs) |  |
| Blood Pressure  mmHg | / mmHg |
| Heart Rate beats/min | beats/min |
| Temperature C | . C |
| Respiration Rate breath/min | breaths/min |
| Oxygen Saturation | %  Not documented |
| Level of  consciousness | Alert  New Confusion  Responsive to Voice  Responsive to pain  Unconscious |
| Method of Ventilation | Room air  Supplement of Oxygen  Other: ________________________ |

| If subject requires oxygen supplementation or a form of ventilation to maintain support respiration, please select type | N/A  O2 supplementation - mask (not high flow)  O2 supplementation - mask (high flow)  O2 supplementation - nasal canulae (not high flow)  O2 supplementation - nasal canulae (high flow)  Non-invasive mechanical ventilation  Mechanical invasive ventilation  ECMO (extracorporeal membrane oxygen) |
| --- | --- |
| For subjects requiring supplemental oxygen, please provide the oxygen flow rate  (l/min) and/or fraction of inspired oxygen (FiO2) (%) | N/A  Oxygen Flow Rate L/min  Fraction of inspired oxygen (Fi02) % |
| For patient requiring non-invasive ventilation, please choose type | N/A  CPAP  BiPAP  Other, specify___________________________ |
| For subjects requiring mechanical ventilation, please provide form of intubation | N/A  Endotracheal tube  Tracheostomy tube |

**Serious Adverse Event Form**

Date of Report: ____ ___/___ ___/___ ___ ___ ___

AKU 20-___ ___ IERC #: 2020/IERC- ___ ___

Protocol Number (if any): _______ Sponsor: _________________

Principal Investigator:

Subject Number: ____________ Subject Initials: _________________ Subject Gender: Subject Date of Birth: ________________

**1. What are you reporting:**  SAE  AESI  Pregnancy

**2. Report type:**  Initial Report  Follow up Report # ____  Final Report

**3. Date site became aware of event:** ___ ___/___ ___/___ ___ ___ ___

**Evaluation of Event**

**4. Event / Reaction:** (key words; e.g. body site, symptoms, severity, treatment)

**5.** a) Date of Onset: ___ ___/___ ___/___ ___ ___ ___

b) Time of Onset (If available): ____ _____ : _____ _____

**6. Criteria for definition as SAE:**

Resulted in Death

Life threatening

In-patient hospitalization or Prolongation

Persistent or significant disability

Congenital anomaly/birth defect

*If there is more than one criterion, choose the most significant one. Seriousness is a regulatory definition and should not be confused with severity.*

**7. Describe Event:** (A summary of signs and symptoms, diagnosis, treatment of event, concurrent treatment, other relevant medical history, including re-challenge details if applicable. Please include the point in the study at which the event occurred.)

**8.** **In the Investigator’s opinion, was the event 9. Action taken with IP?**

**related to the IP?** None

Related Dose temporarily reduced

Not Related Dose reduced

Discontinued temporarily

**10. If related to IP, was this reaction unexpected?** Permanently discontinued

Yes

No

Not Applicable

**11. Did event/reaction abate after stopping drug? 12. Did event/reaction reappear**

**after Re-Introduction of drug?**

Yes Yes

No No

Not Applicable Not Applicable

**13. IMP & Concomitant Medication Information**

| IP | | | | | |
| --- | --- | --- | --- | --- | --- |
| Details (include daily dose(s) & generic name) | Therapy Start Date (dd/mm/yyyy) | Therapy End Date (dd/mm/yyyy) | Date of dose prior to SAE onset  (dd/mm/yyyy) | Route(s) of administration | Indications for Use |
|  |  |  |  |  |  |
|  |  |  |  |  |  |
|  |  |  |  |  |  |
|  |  |  |  |  |  |
|  |  |  |  |  |  |
| **Concomitant drug** | | | | | |
| Details (include daily dose(s) & generic name) | Therapy Start Date (dd/mm/yyyy) | Therapy End Date (dd/mm/yyyy) | Date of dose prior to SAE onset  (dd/mm/yyyy) | Route(s) of administration | Indications for  Use |
|  |  |  |  |  |  |
|  |  |  |  |  |  |
|  |  |  |  |  |  |
|  |  |  |  |  |  |

**14. Have Urgent Safety Measures been implemented?**

Yes

No

Not Applicable

**If yes, please detail below:**

**15. What is the outcome of the SAE?**

Recovered/Resolved. Date of resolution: ___ ___/___ ___/___ ___ ___ ___

Resolved/Recovered with sequalae. Date of resolution: ___ ___/___ ___/___ ___ ___ ___

Continuing

Resulted in Death. Date of death: ___ ___/___ ___/___ ___ ___ ___

Cause of death: ______________________________

Cause obtained from:  Death Certificate Working diagnosis*/ discharge summary*

Unknown

**Contact & Signatures**

Please supply contact details where further information may be obtained:


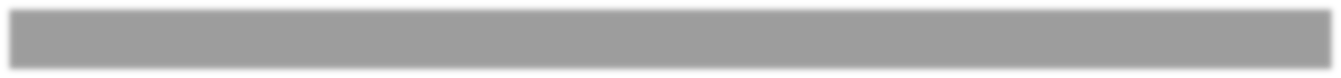

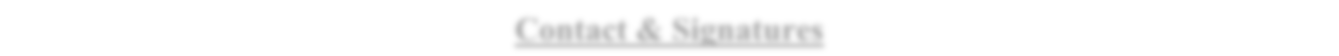

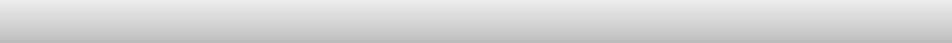

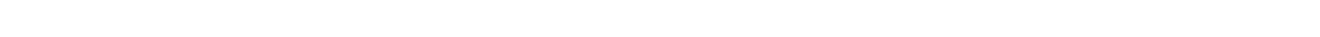


**Name of contact: _____________________**

**Phone number: _______________________**

**Email address: _______________________________________**

**Person completing report, if not PI:**

________________________________ ____________________________ ____________________

Printed name Signature Date

**Principal Investigator:**

________________________________ ____________________________ ____________________

Printed name Signature Date
