# Supplementary material for: A pilot phase Ib/II study of whole-lung low dose radiation therapy (LDRT) for the treatment of severe COVID-19 pneumonia: First experience from Africa
Source: PLoS One. 2022 Jul 1;17(7):e0270594. doi: 10.1371/journal.pone.0270594 (PMC9249221; doi:10.1371/journal.pone.0270594)
Supplement: Attachment — Submitted filename: Response to Reviewers 2 6 22.docx [file pone.0270594.s005.docx]

**A Pilot Phase Ib/II Study of Whole-Lung Low Dose Radiation Therapy (LDRT) For the Treatment of Severe COVID-19 Pneumonia: First Experience from Africa**

Dear Dr Kimple,

We very much appreciate the review of our revised manuscript and the very helpful comments provided by the reviewer. We have responded to each of the comments and revised our manuscript accordingly. The revised manuscript includes the revisions as well as additional references. We thank the reviewers for their comments for which has served to strengthen our submission. We look forward to a positive decision by the editorial team.

Herewith please find our responses to each of the reviewer comments/suggestions:

**Reviewer #1:**

Comment:

Thank you to the authors for resubmitting this manuscript. It has improved substantially and reads well. only two stylistic points to consider:

1. Lines 88-89 - consider citing the other studies here but leave that to author preference
2. Line 306: 'discharge' would be better as past tense 'discharged'.

Response:

The reviewer’s insight and input is much appreciated and we have revised our manuscript and incorporated these suggestions.

Refer to P. 5 Line 88-89 and P. 18 Line 313(previously line 306)

**Reviewer #3:**

Comment:

Nicely written paper, I have some minor comments:

1. Why is no trial registration number reported?

Response:

The reviewer’s comments are well received and we have now included the trial registration number in the manuscript. The trial registration number had been previously submitted via the submission portal during the original submission.

Refer to P. 6 Line 109-110

2. Replace the figures in figure 3 with spaghetti-plots (i.e. individual lines per patient), "dead" or "Discharged" can be different line types but each patient can have a different colour. By doing this with figure 3 it will complement figure 2 (which shows the distribution at each time point) with the individual progress per patient over time.

Response:

We very much appreciate the insightful input from Reviewer # 3 and the suggestion to use a spaghetti plot as opposed to the box plot for Fig. 3. We have followed this advice. However, the small sample size and missing data points for some of the patients makes the spaghetti plot less definitive in demonstrating the upward trend in inflammatory markers in those patients who died. This trend seems to be more apparent when data is summarized into a boxplot for each inflammatory marker. For this reason, we have opted to include the original boxplots as supplementary material.

Refer to Figure 3 Page 14 Line 238-239, 250-255 and Page 18 Line 328-331
